# ﻿The *Leptogenys* Roger, 1861 (Formicidae, Ponerinae) of Hong Kong SAR with additional records from Guangdong, China

**DOI:** 10.3897/zookeys.1202.120214

**Published:** 2024-05-17

**Authors:** Matthew T. Hamer, Jonathan Hon Chung Lee, Cheung Yau Leo Tse, Thiago S. R. Silva, Benoit Guénard

**Affiliations:** 1 School of Biological Sciences, The University of Hong Kong, Kadoorie Biological Sciences Building, Pok Fu Lam Road, Hong Kong SAR, China The University of Hong Kong Hong Kong China; 2 The Hong Kong Biodiversity Museum, The University of Hong Kong, Hong Kong SAR, China The University of Hong Kong Hong Kong China

**Keywords:** Ants, Foo Fighters, Hymenoptera, taxonomy

## Abstract

*Leptogenys* is the most diverse genus of the ant subfamily Ponerinae and is widely distributed across the world’s tropical and subtropical regions. More than 40 species are known from the Oriental realm displaying a wide range of ecologies, although their life history traits remain poorly understood, and new species are frequently discovered. Here, a faunal review of the genus from Hong Kong SAR, southern China is provided. A total of nine species are recorded, with one new species, *Leptogenysgrohli* Hamer, Lee & Guénard, **sp. nov.** described. Ecological and biogeographic information, including new information on reproductive modes for two species are provided with the ergatoids of *L.binghamii* Forel, 1900 and *L.rufida*Zhou et al., 2012 described. Additional records for five of these species within the neighbouring province of Guangdong are also provided. Finally, an illustrated key to species known from Hong Kong is presented, as well as notes on each species’ distribution, ecology, and behaviour. An updated provincial distributional checklist of the *Leptogenys* species of Mainland China and Taiwan is also supplied.

## ﻿Introduction

The ant genus *Leptogenys* is currently recognised as the most diverse genus within Ponerinae, comprising 318 species and 14 subspecies (Bolton 2023). The genus is pantropically distributed, with its greatest diversity encountered within tropical regions, and a limited number of species extending to higher latitudes into warm temperature regions (Janicki et al. 2016; Guénard et al. 2017). Based on a molecular phylogenetic study (Schmidt 2013), the genus is hypothesised to have an Old World origin and to confirm this, a broader understanding of its taxonomic and ecological diversity is paramount. *Leptogenys* species are highly predatory, with epigaeic and leaf-litter foragers that predominantly hunt upon isopods, diplopods, earthworms, termites, earwigs, and other leaf litter invertebrates (Maschwitz et al. 1979; Steghaus-Kovac and Maschwitz 1993, Dejean and Evraerts 1997; Schmidt and Shattuck 2014; Peeters and De Greef 2015; Mizuno et al. 2022). Predatory behaviours range from solitarily foraging, to mass raiding reminiscent of doryline army ants, with a continuum of foraging and recruitment modes between both extremes (Maschwitz et al. 1989; Duncan and Crewe 1994; Janssen et al. 1997; Schmidt and Shattuck 2014; Mizuno et al. 2022). Nests are often ephemeral and typically found within soil, dead wood, or leaf litter, with the whole colony moving to new nest sites frequently (Maschwitz and Mühlenberg 1975; Schmidt and Shattuck 2014), with species of the *processionalis* group even forming temporary bivouacs (Maschwitz et al. 1989). Colonies are often queenless, with reproductive functions performed by ergatoid individuals, and with some species reproducing via a gamergate system (Peeters 1991; Ito 1997; Schmidt and Shattuck 2014). The high species diversity, wide variety of life histories, including varying forms of predation, foraging and reproduction, make *Leptogenys* an ideal taxon for the study of evolution, and behavioural ecology (Dejean and Evraerts 1997).

*Leptogenys* has seen several regional taxonomic treatments in the last few decades, particularly from Asia where Xu and He (2015) provided a revision of the Oriental species and a preliminary regional key. Subsequent work has been produced by Zryanin (2016), Arimoto (2017), Wachkoo et al. (2018), Arimoto and Yamane (2018) for select Oriental species and for species such as the *L.chalybaea* and *L.modiglianii* species groups. Numerous species across the Oriental realm, however, remain undescribed (Arimoto 2017; AntWeb 2024), and many regions remain woefully under-sampled and/or lacking contemporary taxonomic revisions. In China specifically, most of the taxonomic studies for the genus are geographically restricted to a few provinces (e.g., Zhou 2001 and Zhou et al. 2012 for Guangxi, Xu 2000 for Yunnan, and Chen et al. 2024 for Hainan) with the vast majority of regions and provinces of China still lacking local taxonomic revisions for this genus.

Here we review the *Leptogenys* of Hong Kong SAR, one of the most densely populated regions of 6,700 people per square kilometre. Hong Kong, though highly urbanised, comprises surprisingly high biodiversity, including many newly recorded and newly described ant species following intensive sampling from early 90s to the present day (Fellowes 1996; Luo and Guénard 2016; Pierce et al. 2019; Tang et al. 2019; Brassard et al. 2020; Wong and Guénard 2021; Hamer et al. 2023a, Hamer et al. 2023b; Silva et al. 2023; Tang and Guénard 2023). Here, a total of nine *Leptogenys* species are recorded from Hong Kong including four newly recorded species, and one new species to science, *Leptogenysgrohli* sp. nov. Along with the taxonomic accounts, we provide high resolution images, an illustrated dichotomous identification key, and discussions on the morphology as well as the ecology for all species known from Hong Kong. Ergatoid queens of *L.binghamii* and *L.rufida* are also described. Records for five of the overall nine species occurring in Hong Kong are provided for the neighbouring Chinese province Guangdong.

## ﻿Materials and methods

### ﻿Sampling

Most sample collections were performed using leaf litter sampling through Winkler extractors by Dr. John Fellowes between 1993–2002 and members of the Insect Biodiversity and Biogeography Laboratory (IBBL, HKU) between 2014–2023 in Hong Kong. Other sampling methods were conducted during the same periods, including pitfall trapping, baiting and hand collection comprising of nesting locations and general forager collections.

### ﻿Measurements and images

**Figure 1.**
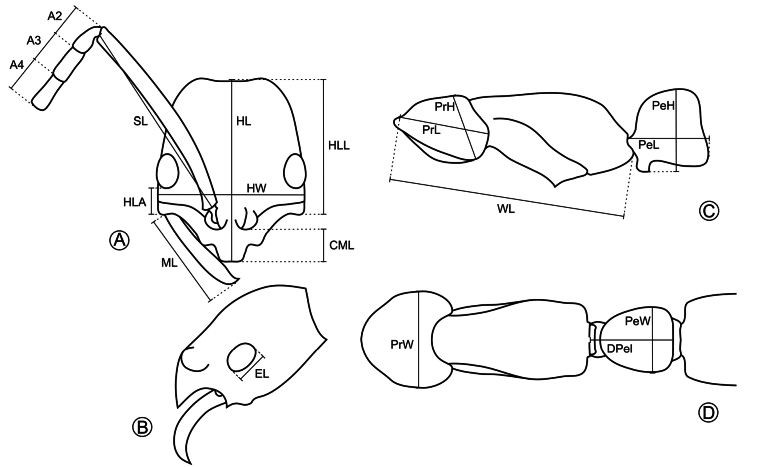
Schematic diagram of the linear measurements used within this study **A** head measurements **B** eye diameter **C** lateral mesosomal and petiole measurements **D** dorsal mesosomal and petiole measurements.

Here we use the core set of linear measurements and indexes broadly used in ant taxonomic studies, as well as specialised measurements suggested by Arimoto and Yamane (2018) (Fig. 1). The lengths of the pedicel (**AII**) and the first two flagellar segments (**AFI–AFII**) are also included here. Measurements for all specimens as well as specimen images were gathered using a DMC5400 Camera attached to a Leica M205C Stereomicroscope and processed in Leica Application Suite (LASX). Based on scaled micrometre calibration, measurements are accurate to 0.01 mm. Image artefacts were removed in Adobe Photoshop with plates produced in Adobe InDesign. Illustrations were made using high-resolution images as base for tracing in Adobe Illustrator.

### ﻿Linear measurements and indices abbreviations

**HL** Head length: the length of head from the anteriormost point of the clypeal lobe to the posteriormost point of the head capsule, measured in full face view.

**HLL** Lateral head margin length: length of head from mandibular base to posteriormost margin of head capsule, measured in full face view.

**HLA** Anterior head length: length of head from mandibular base to anteriormost point of eye, measured in full face view.

**CML** Clypeal median lobe length: length of the clypeal lobe from anteriormost margin to the anterior margin of the torulus, measured in full face view.

**HW** Head width: maximum width of head, excluding eyes, measured in full face view.

**SL** Scape length: diagonal length of scape excluding the neck and basal condyle.

**AII** Antennal segment II length: maximum length of pedicel (antennal segment II), measured in dorsal view.

**AFI** Antennal flagellomere I length: maximum length first flagellar segment, measured in dorsal view.

**AFII** Antennal flagellomere II length: maximum length of second flagellar segment, measured in dorsal view.

**EL** Eye length: the maximum diameter of the compound eye in lateral view.

**ML** Mandibular length: length of mandible from base to apex, measured in full face view.

**PrL** Pronotal length: diagonal length of pronotum measured from anteriormost point of pronotum (excluding neck), to posteriormost margin, measured in lateral view.

**PrH** Pronotal height: height of pronotum from the posterior base of the pronotum to the highest point of pronotum dorsum, measured in lateral view.

**PrW** Pronotal width: maximum width of pronotum, measured in dorsal view.

**WL** Weber’s length: diagonal length of mesosoma from anteriormost point of pronotum (excluding neck) to posteriormost point of propodeal lobe, measured in lateral view.

**PeL** Petiole length: length of petiole from the anteriormost point of the petiole (including petiole peduncle) to the posteriormost point, measured in lateral view.

**PeH** Petiole height: height of petiole from the most ventral margin of the subpetiolar process to the highest point of petiolar dorsal margin, measured in lateral view.

**PeW** Petiole width: maximum width of petiole in dorsal view.

**DpeL** Dorsal petiole length: length of petiole in dorsal view, from anteriormost point of the petiole (including peduncle) to the posteriormost point, measured in dorsal view.

**CI** Cephalic index: HW / HL × 10.

**CLI** Clypeus index: CML / HL × 10.

**SI** Scape index: SL / HW × 10.

**OI** Ocular index: EL / HLL × 10.

**LPI** Lateral petiole index: PeH / PeL × 10.

**DPI** Dorsal petiole index: PeW / DpeL × 10.

### ﻿Depository institution abbreviations

**HKBM** Hong Kong Biodiversity Museum, University of Hong Kong.

**IBBL** Insect Biodiversity and Biogeography Laboratory, The University of Hong Kong.

**SKYC** Seiki Yamane Collection, Kitakyushu Museum of Natural History and Human History, Kitakyushu, Fukuoka, Japa.

**ZRC** Zoological Reference Collection, Lee Kong Chian Natural History Museum, Singapore.

## ﻿Results

### ﻿Key to *Leptogenys* workers of Hong Kong SAR

**Table:** 

1	Anterior clypeal margin terminating in a narrowly convex point (Fig. 2A)	**2**
–	Anterior clypeal margin terminating in a distinct truncation (Fig. 2B)	**4**
2	Pedicel as long as flagellomere I (AII 0.17–0.19; AFI 0.13–0.15) (Fig. 3A); in lateral view, petiole subquadrate, as long as high (LPI 82.61–114.29) (Fig. 3C)	***Leptogenysrufida***
–	Flagellomere I considerably longer than pedicel (AII 0.17–0.24; AFI 0.31–0.39) (Fig. 3B); in lateral view, petiolar node triangular, either longer than high or as long as high (LPI 74.93–97.24) (Fig. 3D)	**3**
3	In full-face view, head lateral margin convex; head dorsum predominantly smooth, with very sparse punctation; eye larger (EL 0.29–0.37)	***Leptogenyspeuqueti***
–	In full-face view, head lateral margin straight to weakly tapering posteriorly; head dorsum densely punctate; eye smaller (EL 0.22–0.27)	***Leptogenysgrohli* sp. nov.**
4	Masticatory margin of mandible with 3–5 teeth (Fig. 4A); head dorsum smooth, with sparse hair bearing punctures only	***Leptogenysstrena***
–	Masticatory margin of mandible edentate, or with a single tooth (Fig. 4B); head dorsum sculpture varying, not as above	**5**
5	Lateral clypeal margin with conspicuous angulate lobes (Fig. 5A); first and second gastral tergites densely sculptured throughout (Fig. 5C)	***Leptogenysbinghamii***
–	Lateral clypeal margin lacking conspicuous angulate lobes (Fig. 5B); first and second gastral tergites smooth and shiny (Fig. 5D)	**6**
6	Petiole almost as long as high in lateral view (LPI 89.2–99.35); dorsal margin with a distinct anterior to posterior curvature (Fig. 6A)	***Leptogenyskraepelini***
–	Petiole as high as long or higher than long in lateral view (LPI 107.12–158.96); dorsal margin flat, lacking conspicuous anterior to posterior curvature (Fig. 6B)	**7**
7	Head dorsum smooth, other than hair bearing punctation; clypeus with a conspicuous median longitudinal carina (Fig. 7A); pronotum entirely smooth	***Leptogenyslaeviterga***
–	Head dorsum with longitudinal costulae; clypeus either entirely lacking median carina or with a weakly produced, inconspicuous carina (Fig. 7B); pronotum variably sculptured	**8**
8	Sides of pronotum smooth, finely rugulose or finely reticulate (Fig. 8A); masticatory margin with a single tooth; generally a smaller species (WL 2.33–2.53)	***Leptogenysdiminuta***
–	Sides of pronotum longitudinally striate (Fig. 8B); masticatory margin without teeth; generally a larger species (WL 2.64–2.87)	***Leptogenyskitteli***

**Figure 2.**
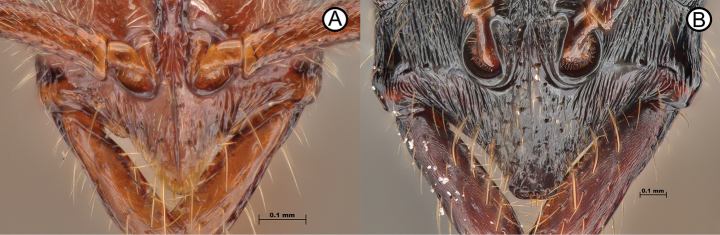
Differing anterior clypeal shapes **A***Leptogenysrufida* (RHL1259) **B***L.kraepelini* (ANTWEB1010120).

**Figure 3.**
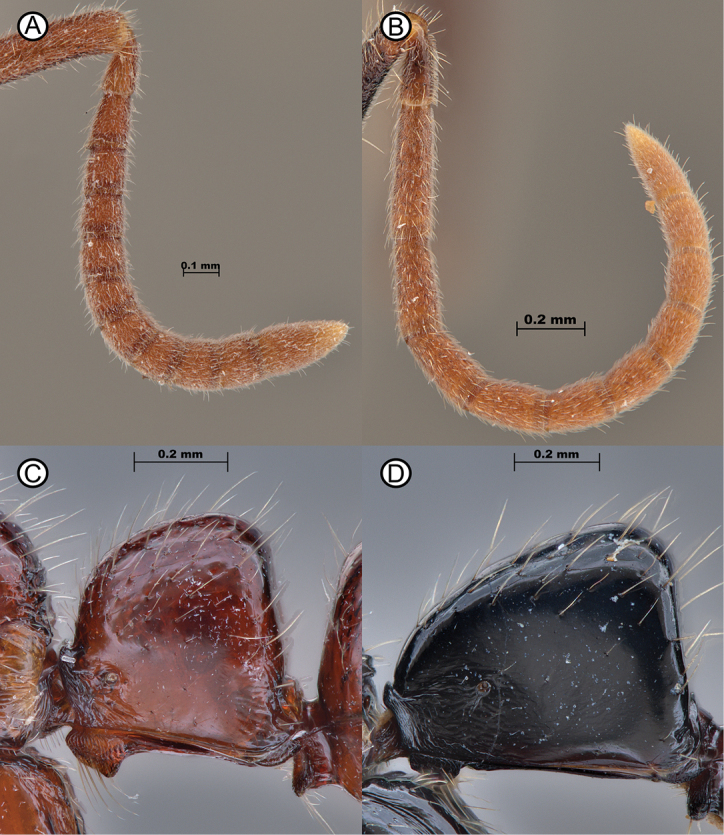
Differences between antennal segment lengths and petiolar shapes **A***Leptogenysrufida* (MBS006585) **B***L.peuqueti* (ANTWEB10096169) **C***Leptogenysrufida* (MBS015251) **D***L.peuqueti* (RHL003347).

**Figure 4.**
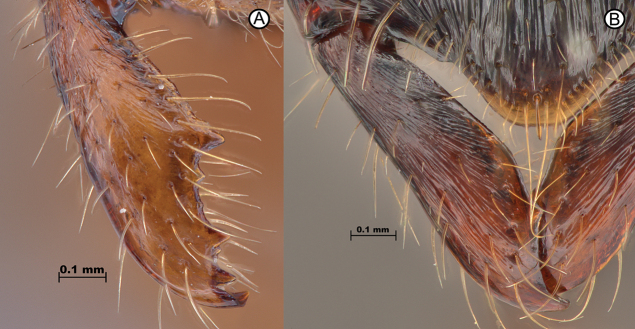
Mandibular masticatory margin differences **A***Leptogenysstrena* (ANTWEB1010114) **B***L.diminuta* (GYOT070).

**Figure 5.**
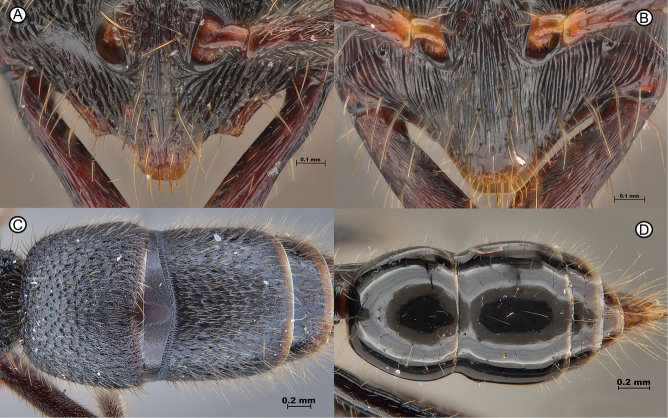
Clypeus lobes of *Leptogenysbinghamii* and *L.diminuta*, as well as tergite sculpture differences between the same species **A***L.binghamii* (RHL01659) **B***L.diminuta* (GYOT070) **C***L.binghamii* (ANTWEB1010113) **D***L.diminuta* (ANTWEB1010183).

**Figure 6.**
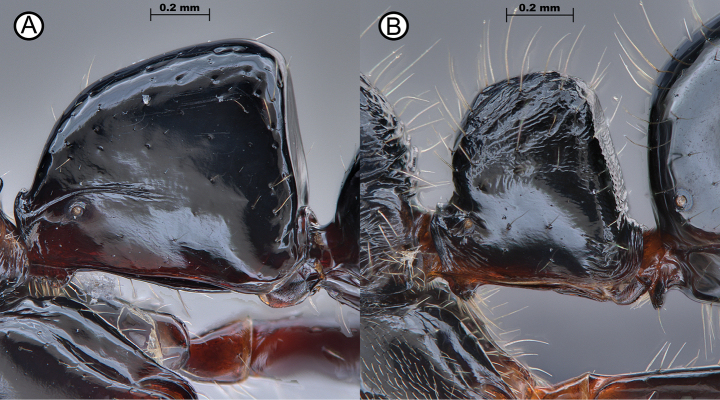
Contrasting petiole shapes between *L.kraepelini* and *L.diminuta***A***L.kraepelini* (ANTWEB1010121) **B***L.diminuta* (ANTWEB1010183).

**Figure 7.**
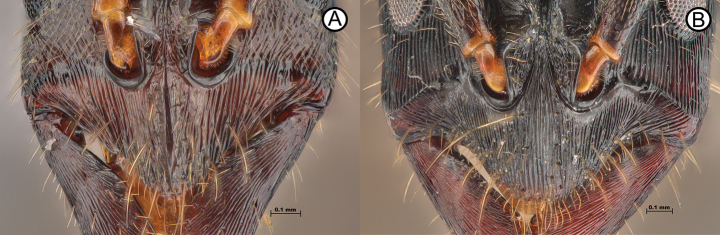
Clypeus differences between *Leptogenyslaeviterga* and *L.kitteli***A***L.laeviterga* (ANTWEB1010142) **B***L.kitteli* (RHL02795).

**Figure 8.**
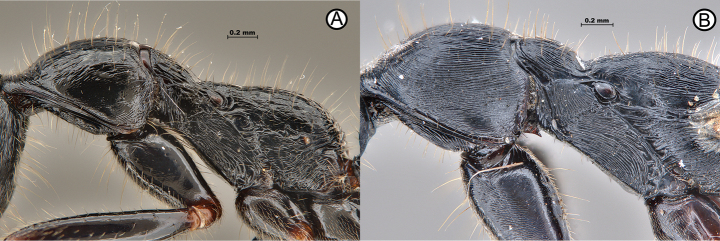
Diverging mesosomal sculpture between *L.diminuta* and *L.kitteli***A***L.diminuta* (ANTWEB1010183) **B***L.kitteli* (PFL1T2W5-1).

### ﻿Taxonomic accounts

#### ﻿ Leptogenys binghamii

Taxon classificationAnimaliaHymenopteraFormicidae

Forel, 1900

5F1A7F9C-49D0-5EBC-8131-7ECD4255FD24

**Figure 9.**
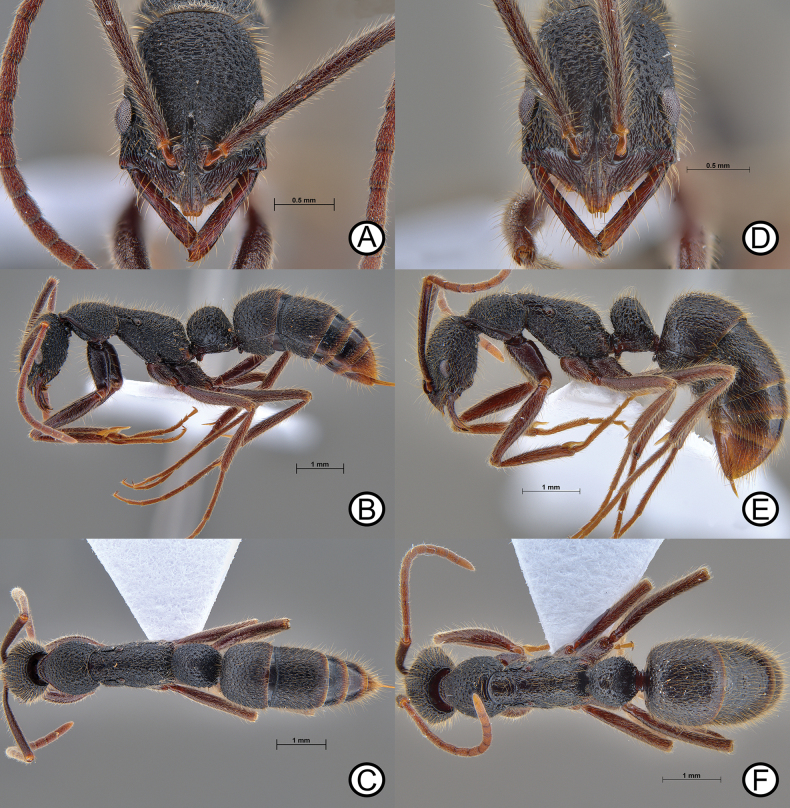
*Leptogenysbinghamii* (ANTWEB1010110) and ergatoid (ANTWEB1010225) **A** worker in lateral view **B** worker in dorsal view **C** worker in head in full face view **D** ergatoid in lateral view **E** ergatoid in lateral view and **F** ergatoid in dorsal view.

Figs 5A, C , 9 , 20A

Leptogenys (Lobopelta) binghamii Forel, 1900f: 310 (w.) Myanmar.
Lobopelta binghamii : Bingham 1903: 58.
Leptogenys (Lobopelta) binghamii : Emery 1911e: 102.

##### Ergatoid description.

With characters of worker but head as wide anteriorly as posteriorly; pronotum wider than remaining mesosoma in dorsal view; mesosoma stout and robust, less elongated as worker; petiole nodiform, distinctly higher than long in lateral view; about as wide as long in dorsal view. Metasomal segments III–VII enlarged, segment III distinctly wider than petiole. Same colour as the worker.

##### Measurements.

Worker (*n* = 14); HL 1.66–1.84; HLL 1.31–1.47; HLA 0.24–0.3; HW 1.18–1.33; CML 0.31–0.39; SL 1.94–2.13; AII 0.21–0.33; AFI 0.43–0.51; AFII 0.37–0.41; EL 0.31–0.36; ML 0.84–1.03; PrL 1.04–1.25; PrH 0.77–1.13; PrW 1.04–1.2; WL 2.76–3.1; PeL 0.8–1.04; PeH 0.95–1.18; PeW 0.77–0.92; DPL 0.87–0.95; CI 69.36–74.97; CLI 17.58–22.49; SI 156.26–166.48; OI 21.98–26.37; LPI 104.64–124.38; DPI 84.67–98.51.

Ergatoid paratype (*n* = 1): HL 1.67; HLL 1.29; HLA 0.31; HW 1.25; CML 0.39; SL 1.77; AII 0.23; AFI 0.39; AFII 0.31; EL 0.34; ML 0.96; PrL 1.03; PrH 0.76; PrW 1.04; WL 2.56; PeL 0.75; PeH 1.06; PeW 0.62; DPL 0.83; CI 74.7; CLI 23.33; SI 141.31; OI 26.49; LPI 142.49; DPI 73.98.

##### Morphological variation.

Little to no variation, other than morphometric values, were detected in the specimens examined from Hong Kong.

##### Comparative notes.

*Leptogenysbinghamii* is a relatively large, highly sculptured species, with distinctly linear mandibles, small anteriorly positioned eyes and highly angulated lateral clypeal lobes which makes it recognisable within the *Leptogenys* found in Hong Kong. Within the wider *Leptogenys* fauna of the Indomalayan region, *L.binghamii* might be mistaken for *L.punctiventris* (Mayr, 1879) and *L.yandii* (Xu & He, 2015). *Leptogenysbinghamii* is distinguishable from *L.yandii* by antennal flagellomere I longer than antennal flagellomere II, the longer scape, and larger overall size (5.2–5.7 vs 9–10 mm total length for *L.yandii* and *L.binghamii*, respectively; Xu and He 2015). *Leptogenysbinghamii* can be differentiated from *L.punctiventris* by the smaller eyes, the first gastral tergite being highly punctate, lacking any smooth and shiny regions as well as total size (5–6 vs 9–10 mm total size for *L.punctiventris* and *L.binghamii*, respectively; Xu and He 2015). Both *L.punctiventris* and *L.yandii* are not recorded from Hong Kong. With these species presenting more meridional and occidental distributions within Asia, respectively.

##### Distribution.

This species is known from Myanmar (type locality), India (Assam and Meghalaya), China (Guangxi, Yunnan, and Hong Kong), and Vietnam (Janicki et al. 2016; Guénard et al. 2017). There are no records from Guangdong, Hainan, or Macao, but its presence is expected in the first two Chinese provinces with more sampling efforts. Its presence in Macao is less likely due to the level of urbanisation and isolation of natural areas for this species to occur. The first record of *L.binghamii* in Hong Kong was Fellowes (1996), here we provide additional new records across the eastern limits of its range.

##### Ecology.

In Hong Kong, colonies have been found within decaying wood and underneath stones, predominantly within Feng Shui Woods, secondary forests and shrubland. Colonies appear relatively small, with one partial colony collection including 23 workers, 1 male, 5 cocoons, 4 larvae, and 2 eggs (MTH collection code MTH163). One ergatoid queen was extracted from a partial colony collection (MTH268). Specimens are known from pitfall traps, leaf litter, and hand collection events. This species is suspected to be a solitary foraging species, with no group hunting yet observed. However, unless nesting sites are located, *L.binghamii* is rarely observed diurnally and is therefore suspected to be predominately a nocturnal foraging species. A second colony, kept in captive colony consisted of 24 workers and one ergatoid queen. Workers fed upon isopod prey and showed no interest in cockroaches, millipedes, and termites. In Hong Kong, the species reaches the northern limit of its distribution range with all records found under 500 m a.s.l.. Further sampling is nonetheless required to confirm if the species can colonize higher and cooler elevations.

##### Material examined.

Worker (*n* = 52): China • 1 worker; Hong Kong SAR, Ng Fai Tong, East Central New Territories; 23 Aug. 1996; J.R.Fellowes leg.; HKBM MBS015249. • 3 workers; Hong Kong SAR, Pak Ngau Shek; 24 Oct. 1996; J.R.Fellowes leg.; HKBM MBS006888, MBS015265, MBS06604. • 1 worker; Hong Kong SAR, Yau Ping San UK, East Central NT; 3 Oct. 1996; J.R.Fellowes leg.; HKBM MBS015263. • 2 workers; Hong Kong SAR, Kadoorie Farm Botanical Garden; 22.4302, 114.1192; 280 m a.s.l.; 14 Sep. 2015; R.H.Lee leg.; Secondary forest, Pitfall trap; IBBL RHL02700 RHL02717. • 1 worker; Hong Kong SAR, Lion Rock; 22.35805, 114.17699; 150 m a.s.l.; 13 Jul. 2015; R.H.Lee leg.; Secondary forest, Pitfall trap; IBBL RHL01659. • 1 worker; Hong Kong SAR, Pak Tam Chung; 22.401, 114.33; 130 m a.s.l.; 5 Jun. 2015; R.H.Lee leg.; Shrubland, Pitfall trap; IBBL RHL02373. • 1 worker; Hong Kong SAR, Shing Mun; 22.39678, 114.1531; 240 m a.s.l.; 14 May. 2015; R.H.Lee leg.; Feng Shui Forest, Pitfall trap; IBBL RHL000350. • 1 worker; Hong Kong SAR, Pok Fu Lam; 22.267, 114.1438; 248 m a.s.l.; 7 Jul. 2016; M.Pierce leg.; Secondary forest, Bait trap; IBBL ANTWEB1009150. • 1 worker; Hong Kong SAR, Lion Rock; 22.357, 114.17504; 140 m a.s.l.; 15 Aug. 2017; R.H.Lee leg.; Secondary forest, Pitfall trap; IBBL RHL03531A. • 1 worker; Hong Kong SAR, Tai Po Kau; 22.42285, 114.18082; 200 m a.s.l.; 6 Jun. 2017; R.H.Lee leg.; Secondary forest, Pitfall trap; IBBL RHL03550. • 4 workers; Hong Kong SAR, Ngong Ping SSSI; 22.25364, 113.90129; 438 m a.s.l.; 24 May. 2022; M.T.Hamer leg.; Secondary forest, Nest ex. Decay wood; IBBL ANTWEB1010110, ANTWEB1010111, ANTWEB1010112, ANTWEB1010113. • 1 worker; Hong Kong SAR, Tai Mo Shan; 22.40549, 114.16352; 468 m a.s.l.; 19 May. 2023; L.Xuan leg.; Secondary forest, Hand collection; IBBL ANTWEB1010125. • 5 workers; Hong Kong SAR, Tai Mo Shan; 22.40403, 114.1069; 471 m a.s.l.; 26 Aug. 2023; M.T.Hamer leg.; Secondary forest, un. Rock; IBBL MTH163. • 5 workers; Hong Kong SAR, Lantau; 22.31701, 114.0173; 179 m a.s.l.; 29 Aug. 2023; M.T.Hamer leg.; Young secondary, ex. Decaying log; IBBL MTH221. • 1 worker; Hong Kong SAR, Lantau; 22.31701, 114.01733; 179 m a.s.l.; 29 Aug. 2023; M.T.Hamer leg.; Young secondary forest, Nest ex. Decay log; IBBL ANTWEB1010173. • 12 workers; Hong Kong SAR, Tai Mo Shan; 22.40452, 114.10645; 490 m a.s.l.; 27 Sep. 2023; M.T.Hamer leg.; Secondary forest, ex. Soil; IBBL MTH563. • 1 worker; Hong Kong SAR, Tai Mo Shan; 22.40549, 114.16352; 468 m a.s.l.; 19 May. 2023; L.Xuan leg.; Secondary forest, Hand collection; IBBL ANTWEB1010125.

***Paratype*** ergatoid (*n* = 1): China • 1 ergatoid; Hong Kong SAR, Tai Mo Shan; 22.40403, 114.10691; 470 m a.s.l.; 3 Sep. 2023; M.T.Hamer leg.; secondary forest, ex. Decaying log; IBBL ANTWEB1010225.

#### ﻿ Leptogenys diminuta

Taxon classificationAnimaliaHymenopteraFormicidae

(F. Smith, 1857)

03698FD9-2CFE-55EF-B4F3-06095D6D354E

Figs 4B. 5B, D. 6B, 8A, 10, 20B

Ponera diminuta Smith, 1857a: 69 (w.) Borneo (East Malaysia: Sarawak).
Lobopelta diminuta : Mayr 1862: 734.
Leptogenys diminuta : Emery 1895: 461.
Leptogenys diminuta bismarckensis [senior synonym] Forel, 1901c: 7 (w.) New Guinea: Wilson 1958c: 118; Bolton 1995b: 231; Zhou 2001a: 43. Of Leptogenysdiminutadeceptrix Forel, 1901m: 46 (w.): Xu and He 2015: 138. Of Leptogenysferox Smith, 1865a: 70 (w.) Indonesia: Wilson 1958c: 118; Bolton 1995b: 231; Zhou 2001a: 43. Of Leptogenyshodgsoni Forel, 1900f: 308 (w.) Myanmar: Xu and He 2015: 144. Of Leptogenysdiminutapalliseri Forel, 1900f: 307 (w.m.) India: Xu and He 2015: 138. Of Leptogenysdiminutasantschii Mann, 1919: 299 (w.eq.m.) Solomon Is: Wilson 1958c: 118; Bolton 1995b: 231; Zhou 2001a: 43. Of Leptogenysdiminutasarasinorum Forel, 1900f: 307 (w.) Sri Lanka: Xu and He 2015: 138. Of Leptogenyssimillima Smith, 1860b: 104 (w.) Indonesia: Roger 1863b: 19; Mayr 1863a: 428; Bolton 1995b: 231; Zhou 2001a: 43. Of Leptogenysdiminutastitzi Viehmeyer, 1934c: 310: Wilson 1958c: 118; Bolton 1995b: 231; Zhou 2001a: 43. Of Leptogenysdiminutastriatula Emery, 1895m: 461 (w.) Myanmar: Xu and He 2015: 138. Of Leptogenysdiminutawoodmasoni Forel, 1886d: 246 (w.) India: Xu and He 2015: 138. of Leptogenysdiminutayarrabahna Forel 1915b: 29 (w.m.) Australia: Taylor 1988: 34; Bolton 1995b: 231; Zhou 2001a: 43.

##### Measurements.

Worker (*n* = 10): HL 1.53–1.62; HLL 1.15–1.33; HLA 0.34–0.39; HW 1.09–1.17; CML 0.36–0.41; SL 1.56–1.72; AII 0.22–0.225; AFI 0.29–0.34; AFII 0.23–0.32; EL 0.3–0.36; ML 0.67–0.83; PrL 0.85–0.99; PrH 0.57–0.72; PrW 0.81–0.89; WL 2.32–2.53; PeL 0.49–0.66; PeH 0.65–0.73; PeW 0.4–0.48; DPL 0.42–0.5; CI 70.01–74.19; CLI 23.53–26.32; SI 137.99–155.25; OI 24.21–27.27; LPI 107.12–135.85; DPI 84.24–103.9.

Ergatoid (*n* = 1): HL 1.59; HLL 1.29; HLA 0.39; HW 1.18; CML 0.43; SL 1.63; AII 0.25; AFI 0.32; AFII 0.24; EL 0.34; ML 0.83; PrL 0.94; PrH 0.66; PrW 0.89; WL 2.61; PeL 0.56; PeH 0.7; PeW 0.47; DPL 0.47; CI 74.15; CLI 26.66; SI 137.82; OI 26.43; LPI 124.73; DPI 99.57.

##### Morphological variation.

Studied specimens showed clear variation in dorsal head sculpturing, with the shape of concentric costulae medially ranging from broadly curved to angulate, and the regularity of costulae varying subtly among specimens. Additionally, we observed the presence of smooth patches in the middle of the head, with varying sizes among certain specimens; when present, these patches were between the concentric costulae. The dorsal margin of the node is completely foveate in most specimens, but fine costulae was observed in the ventral margin of the petiole of one specimen. Fine and faint costulae was also observed on the metapleuron of one specimen, contrary to the general pattern of rugulose sculpture found in other specimens of *L.diminuta*. *Leptogenysdiminuta* is known to be a highly morphologically variable species, both on a wider geographical scope, as well as within Hong Kong. This is also reflected through the complex taxonomic history of this species and the numerous associated subspecies, either considered as valid or synonyms. Given such morphological diversity, *L.diminuta* is likely a species complex with numerous cryptic species found across its distribution (Wilson 1958).

##### Comparative notes.

*Leptogenysdiminuta* resembles and is frequently confused with *L.kitteli*. However, *L.diminuta* can be readily separated from *L.kitteli* in Hong Kong based upon the often smooth to finely reticulate pronotum, the presence of a single tooth on the mandibular masticatory margin, as well as its much smaller size relative to *L.kitteli*.

##### Distribution.

A very wide ranging species, or species complex, known from numerous regions across Asia and Oceania, including the Australasian region (Australia, New Guinea, Solomon Islands), the Indomalayan region (Bangladesh, Borneo, mainland China [Guangdong (additional records provided here), Fujian, Hainan, Guangxi, Hunan, Hong Kong], India, Indonesia, Lanka, Laos, Malaysia, Myanmar, Nepal, Philippines, Singapore, Sri Lanka, Taiwan, Thailand, and Vietnam), as well as the Palaearctic region [part of southwestern China (Sichuan)] (Wilson 1958; Bakhtiar and Chiang 2010;; Xu and He 2015; [Bibr B5][Bibr B20]; Guénard et al. 2017). The absence of this species from various countries and provinces (e.g., Cambodia and Guizhou, China) likely reflects lack of sampling efforts.

##### Ecology.

**Figure 10.**
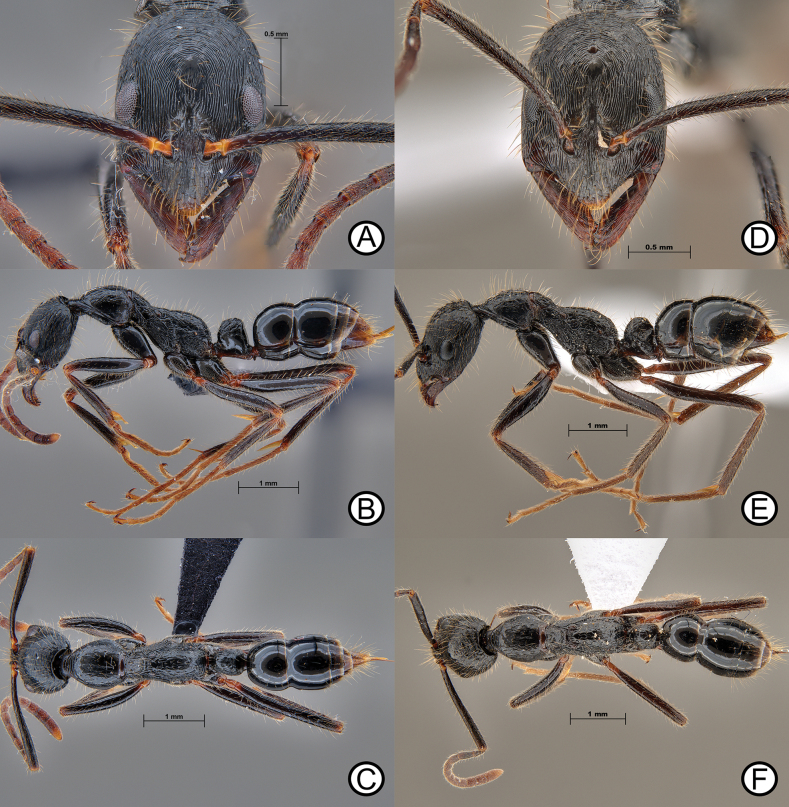
*Leptogenysdiminuta* worker (ANTWEB1010183) and *L.diminuta* ergatoid (ANTWEB1010180) **A** worker specimen in lateral view **B** worker specimen in dorsal view **C** worker specimen in full face view **D** ergatoid in lateral view **E** ergatoid in lateral view and **F** ergatoid in dorsal view.

*Leptogenysdiminuta* is a group-hunting species, forming long columns of workers that move through their habitat in search of prey. Colonies are moderately populous with 200–400 individuals per nest and move nesting locations throughout the year (Ito 1997). Nest locations in Hong Kong have included rotting logs, underneath rocks and within leaf litter. The species has ergatoids (Fig. 10D–E) and likely disperses through colonial budding (Ito 1997; Ito and Ohkawara 2000). Records are predominantly known from young to old growth secondary forests, and similar to *L.peuqueti*, it is one of the most frequently encountered *Leptogenys* in Hong Kong.

##### Material examined.

Workers (*n* = 67): China • 1 worker; Hong Kong SAR, Sunset Peak; 25 Jul. 1992; J.R.Fellowes leg.; IBBL MBS006900. • 3 worker; Hong Kong SAR, Kadoorie Farm; 10 Aug. 1996; J.R.Fellowes leg.; IBBL MBS006880, MBS006881, MBS006882. • 1 worker; Hong Kong SAR, Lantau, Tei Tong Tsai; 5 Nov. 1996; J.R.Fellowes leg.; HKBM MBS015302. • 3 workers; Guangdong Prov., Gu Tian; 10 Apr. 1997; J.R.Fellowes leg.; HKBM MBS006883, MBS006884, MBS006885. • 2 workers; Hong Kong SAR, The Peak; 27 Jun. 1999; SK.Yamane leg.; SKYC ANTWEB1010191, ANTWEB1010192. • 1 worker; Hong Kong SAR, Victoria Park; 27 Jun. 1999; SK.Yamane leg.; SKYC MBS006607. • 1 worker; Hong Kong SAR, Kadoorie Farm Botanical Garden; 6 Sep. 1999; J.R.Fellowes leg.; HKBM MBS015275. • 1 worker; Hong Kong SAR, Kadoorie Farm Botanical Garden; 448–480 m a.s.l.; 21 Sep. 1999; J.R.Fellowes leg.; HKBM MBS015282. • 1 worker; Hong Kong SAR, The Peak; 10 Sep. 2000; SK.Yamane leg.; SKYC ANTWEB1010193, ANTWEB1010195, ANTWEB1010196, ANTWEB1010197. • 1 worker; Hong Kong SAR, Kadoorie Farm Botanical Garden; 22.42947, 114.12145; 350 m a.s.l.; 3 Jul. 2011; P.S.Ward leg.; Secondary forest, Hand collection; IBBL ANTWEB1010181. • 1 worker; Hong Kong SAR, Lin Fa Shan; 22.39963, 114.09654; 423 m a.s.l.; 18 Jul. 2015; T.Tsang leg.; Winkler leaf litter Ex; IBBL TT01320. • 1 worker; Hong Kong SAR, Tai Lam; 22.3952, 114.09072; 440 m a.s.l.; 26 Oct. 2015; R.H.Lee leg.; Shrubland, Hand collection; IBBL RHL01371. • 1 worker; Hong Kong SAR, Ng Tong Chai; 22.42415, 114.13185; 420 m a.s.l.; 26 Jun. 2016; R.H.Lee leg.; Secondary forest, Hand collection; IBBL RHL02822. • 1 worker; Hong Kong SAR, Lung Fu Shan; 22.2758, 114.13546; 260 m a.s.l.; 30 Jun. 2016; R.H.Lee leg.; Secondary forest, Hand collection; IBBL RHL-SIA-008. • 1 worker; Hong Kong SAR, Clear Water Bay; 22.2918, 114.30208; 114 m a.s.l.; 19 Jul. 2016; M.Pierce leg.;, ground forager; IBBL ANTWEB1017020. • 1 worker; Hong Kong SAR, Tai Po; Lam Tseun; 22.43449, 114.11728; 171 m a.s.l.; 30 Aug. 2016; M.Pierce leg.; forest, Hand collection; IBBL ANTWEB1009148. • 1 worker; Hong Kong SAR, Tai Po; Lam Tseun; 22.43449, 114.11728; 171 m a.s.l.; 30 Aug. 2016; M.Pierce leg.; forest, Hand collection; IBBL ANTWEB1009378. • 1 worker; Hong Kong SAR, Lung Fu Shan; 22.2811, 114.1369; 150 m a.s.l.; 5 Oct. 2016; B.Worthington leg.;, Hand collection (night); IBBL BMW02340. • 1 worker; Hong Kong SAR, Tsing Yi; 22.3405, 114.1003; 300 m a.s.l.; 12 Feb. 2017; R.H.Lee leg.; Open rock, general forager; IBBL RHL003394. • 1 worker; Hong Kong SAR, Hatton Road; 22.2792, 114.1365; 237 m a.s.l.; 12 Feb. 2022; M.T.Hamer leg.; Young secondary, Hand collection; IBBL ANTWEB1010183. • 1 worker; Hong Kong SAR, Pok Fu Lam; 22.25988, 114.13944; 213 m a.s.l.; 13 Jun. 2022; M.T.Hamer leg.;, Hand collection; IBBL ANTWEB1010184. • 3 workers; Hong Kong SAR, Ngong Ping; 22.25298, 113.90457; 455 m a.s.l.; 4 May. 2023; M.T.Hamer leg.; Secondary forest, Hand collection; IBBL ANTWEB1010128, ANTWEB1010129, ANTWEB1010130. • 1 worker; Hong Kong SAR, Wong Lung Hang; 22.26368, 113.95375; 400 m a.s.l.; 11 May. 2023; M.T.Hamer leg.; Young secondary forest, Hand collection; IBBL ANTWEB1010133, ANTWEB1010134. • 1 worker; Hong Kong SAR, Yi O; 22.22342, 113.84714; 13 m a.s.l.; 20 Jun. 2023; M.T.Hamer leg.; Young secondary forest, Winkler leaf litter Ex; IBBL ANTWEB1010126, ANTWEB1010127. • 1 worker; Hong Kong SAR, Ngong Ping SSSI; 22.25252, 113.90662; 455 m a.s.l.; 29 Jun. 2023; M.T.Hamer leg.; Secondary Forest, Hand collection; IBBL ANTWEB1010124. • 2 workers; Hong Kong SAR, Tai Mo Shan; 22.40403, 114.1069; 471 m a.s.l.; 22 Aug. 2023; C.Y.L.Tse leg.; Secondary forest, ex. Nest; IBBL MTH243. • 1 worker; Hong Kong SAR, Tai Mo Shan; 22.40384, 114.10549; 491 m a.s.l.; 26 Aug. 2023; C.Y.L.Tse leg.; Secondary forest, Gen. forager; IBBL MTH80, MTH242. • 4 workers; Hong Kong SAR, Tai Mo Shan; Kap Lung Forest Trail; 22.41084, 114.10423; 450 m a.s.l.; 3 Sep. 2023; M.T.Hamer leg.; Secondary forest, ex.decaying log; IBBL MTH222. • 5 workers; Hong Kong SAR, Nei Lak Shan; 22.26524, 113.9064; 500 m a.s.l.; 6 Sep. 2023; M.T.Hamer leg.; Young Secondary Forest, Hand coll. in trail; IBBL MTH303. • 5 workers; Hong Kong SAR, Tai Mo Shan; 22.40452, 114.10645; 490 m a.s.l.; 27 Sep. 2023; M.T.Hamer leg.; Secondary forest, un. rock; IBBL MTH574.

Ergatoid: China • 1 ergatoid; Hong Kong SAR, Tai Mo Shan; Kap Lung Forest Trail; 22.40422, 114.10591; 470 m a.s.l.; 22 Aug. 2023; C.Y.L.Tse leg.; Nest ex.; IBBL ANTWEB1010180.

#### ﻿ Leptogenys grohli

Taxon classificationAnimaliaHymenopteraFormicidae

Hamer, Lee & Guénard sp. nov.

898695E9-B576-5AF2-9C9B-E285CCED4B7E

https://zoobank.org/12FD252A-BAE4-488A-80F7-C5EAA46972D0

**Figure 11.**
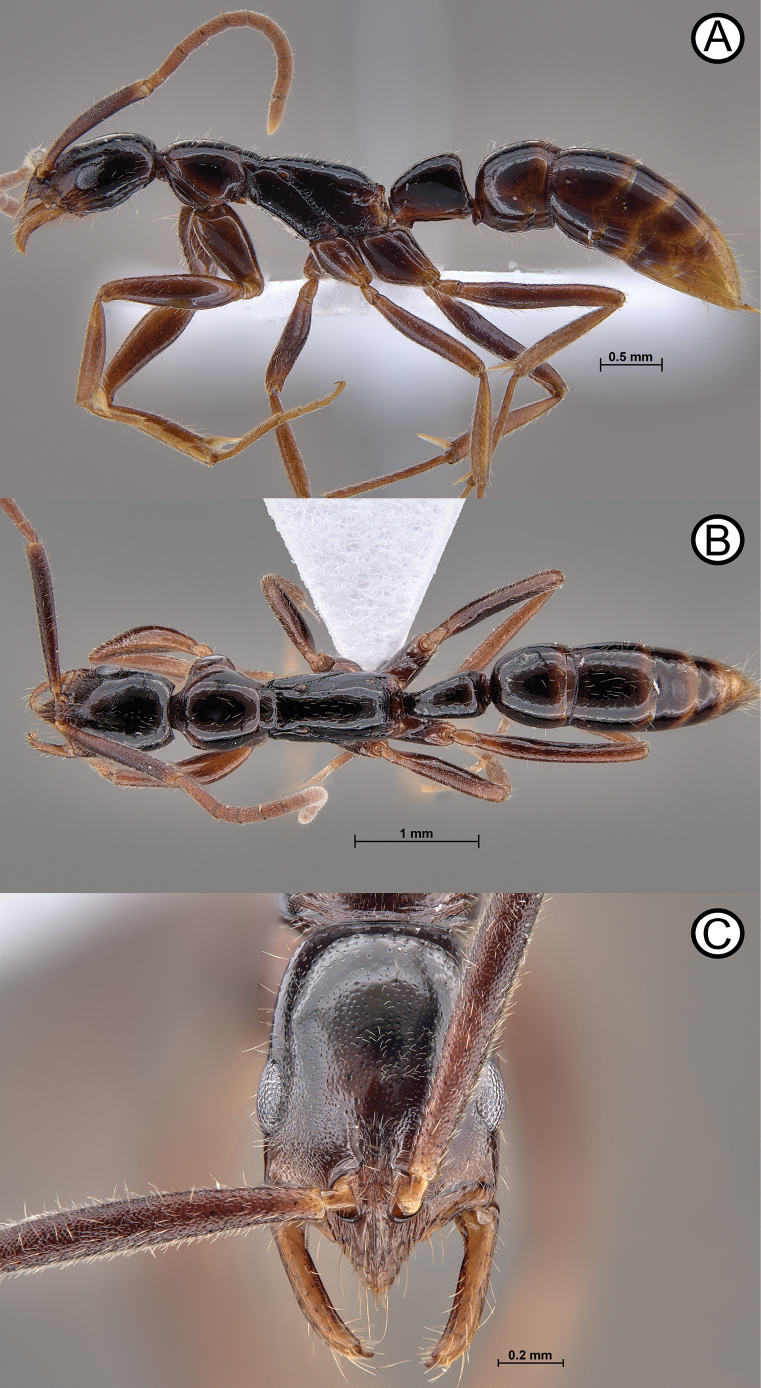
*Leptogenysgrohli* holotype (ANTWEB1010093) **A** lateral view **B** dorsal view **C** head in full face view.

**Figure 12.**
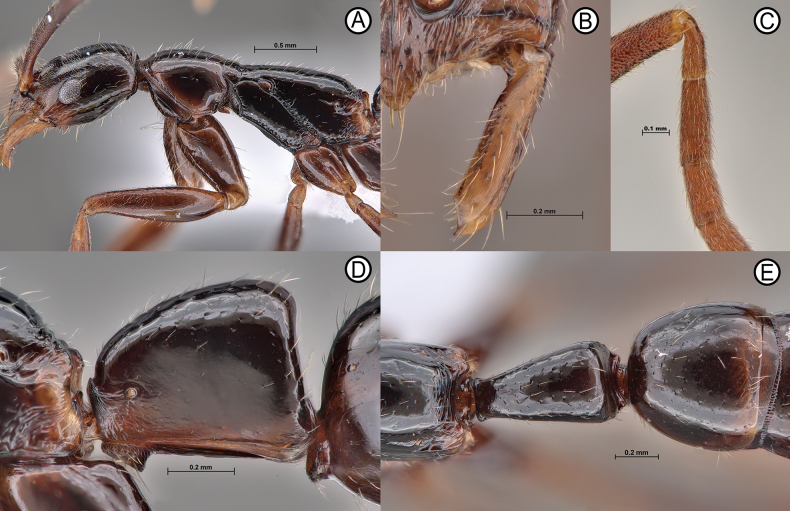
*Leptogenysgrohli* sp. nov holotype (all images are ANTWEB1010093, other than **C** ANTWEB1010092) close up sections **A** close up of head and mesosoma in lateral view **B** close up of right hand mandible in full face view **C** close up of right antennal scape in full anteroventral view **D** close up of petiole in lateral view **E** close up of propodeum, petiole and first gastral tergite in dorsal view.

**Figure 13.**
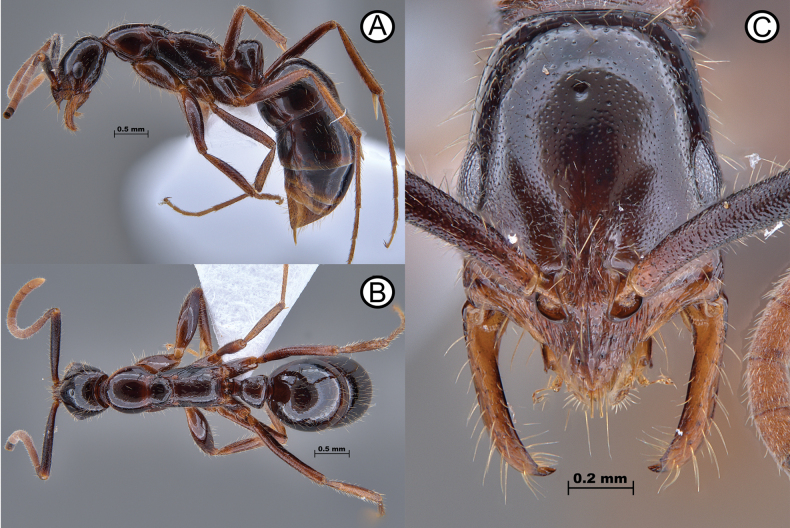
*Leptogenysgrohli* ergatoid queen (ANTWEB1010094) **A** lateral view **B** dorsal view **C** head in full face view.

Figs 11 , 12 , 13 , 20C

##### Diagnosis.

Head isosceles trapezoid, longer than wide; mandible linear, basal margin longer than masticatory margin; basal margin edentate, masticatory margin with one proximal and one apical tooth. Scape extending beyond posterior head margin by three tenths of its length; antennal flagellomere I longer than pedicel and antennal flagellomere II. Promesonotal articulation and notopropodeal sulcus present. Propodeum with a pair of broadly rounded, posterior facing cuticular lobes. In lateral view, petiole slightly longer than high, trapezoidal in shape with posterior margin higher than anterior margin; dorsum subtly convex, curving gradually downwards anteriorly. Head dorsum between eye, clypeus, and frontal lobe with numerous and dense hair-bearing micro-punctulae. Mesosomal dorsum smooth and shining with large, sparse punctures. Anteriormost point clypeal margin with a pair of stout, tubular peg-like setae and two decumbent hairs adjacent to the peg-like setae.

##### Description.

***Head.*** In full face view, head isosceles shaped as trapezoid, longer than wide, widest at mandibular insertion; lateral margin subtly convex and subtly widening beyond eye to posterior margin; posterolateral corner of head blunt; posterior margin of head straight. Mandible linear, basal margin longer than masticatory margin; distinct angle between basal and masticatory margin present; basal margin weakly sinuous and edentate, masticatory margin with one proximal (formed by angle between basal and masticatory margin) and one apical tooth; gap formed between clypeus and mandible when closed. In lateral view, masticatory margin of mandible curves ventrad at apex; mandibular groove present, beginning 1/3 the width of the mandible from base, terminating at ventrolateral mandible apex. Mandalus ovoid, visible at base of mandible when closed. In full face view, anterior clypeal lobe broadly triangular, extending into intermandibular space; 1/5 of head length; terminating in an acute convex point; lateral clypeal margin gradually curving to lateral head margins with inconspicuous lobes; longitudinal clypeal carina present and conspicuous. Frontal lobes small, 2/3 of antennal condyle visible. Frontal groove short, terminating at anterior eye margin. Occipital carina present, extending to ventral surface meeting at an acute angle ventrally. Eye convex, located dorsolaterally in full face view; interrupting lateral head outline; length 1/4 of lateral head length; maximum number of ommatidia across maximum eye length 14 or 15. Antennae with ten flagellar segments; scape long, extending beyond posterior head margin by 3/10 of its length; scape reaching maximum width medially; antennal flagellomere I longer than pedicel and antennal flagellomere II; apical most flagellomere tapering to a point. Hypostomal teeth present; in ventral view, located laterally, adjacent to mandibular base. Palp formula 4:4 (one worker dissected).

***Mesosoma.*** In lateral view, promesonotum feebly to distinctly convex; promesonotum higher than propodeum; propodeal dorsal outline straight to feebly convex; dorsal surface longer than declivitous face; angle between dorsal and declivitous face gradually curving. In dorsal view, pronotum wider than long; pronotum wider than maximum propodeal width. Prosternal process present, anteriorly convex, and posteriorly acutely angled. Promesonotal articulation present but feebly impressed. Mesonotum wider than long. Notopropodeal sulcus present, impressed, with longitudinal cross-ribs. Angle between dorsal and lateral surfaces smoothly curved. Posterolateral propodeum at spiracular height with broadly rounded lobe that extend posteriorly; propodeal declivitous surface concave. In lateral view, mesometapleural suture deeply impressed, with cross-ribs; mesopleural margin of suture distinctly marginated. Mesopleuron not visibly divided into anespiternum and katerpisternum. Propodeal spiracle circular, located above midline of lateral propodeum surface within concave cuticular depression anterodorsally. Metapleural gland bulla circular. Distance between metapleural gland and spiracle greater than spiracle diameter. Legs long and slender; tarsomere one with concave cleaning comb basally. Mesotibia with one pectinate and one simple spur; simple spur 1/2 length of pectinate spur. Metatibia with one long pectinate spur and one simple spur; simple spur ~ 1/3 the length of the pectinate spur. All tarsal claws pectinate.

***Metasoma.*** In lateral view, petiole slightly longer than high, trapezoidal in shape with posterior margin higher than anterior margin; dorsum subtly convex, curving anteriorly; anterior face straight, ~ 1/2 as high as of posterior face. Peduncle absent. Spiracle small and circular, located laterally on anteromedial surface. In dorsal view, petiole distinctly longer than wide; posterior width twice the anterior width. Subpetiolar process present, located anteroventrally; process triangular with posterior margin longer than anterior margin, posteroventral angle acute. Semi-circular prora lobe present, pointed ventrally. Angle between anterior and dorsal surface of abdominal tergite III broadly rounded in lateral view. Abdominal tergite III widest posteriorly in dorsal view. Cinctus with numerous short, longitudinal cross-ribs. Sting present.

***Sculpture.*** Mandibular dorsum with faint longitudinal striations across whole length. Clypeal dorsum laterally smooth, coarsely striate medially, with scattered hair-bearing punctae. Head dorsum between eye, clypeus, and frontal lobe with numerous, dense hair-bearing punctulae; punctations become larger and sparse posteriorly; ventrolateral head surface mostly smooth and shining with widely spaced hair-bearing punctations. Antennal segments with numerous, dense punctulae, lacking smooth regions. Mesosomal dorsum mostly smooth with large, sparse punctures; distance between punctures much larger than their diameter. Propodeal declivity with conspicuous transverse striations that do not extend to propodeal dorsum; posterior portion of declivity smooth. Lateral surface of pronotum mostly smooth and shining with large, sparse punctures. Mesopleuron anteroventral and ventral margins with short striations; striations crossing mesopleuron width ventrally; mesopleuron mostly smooth and shining with sparse punctures. Lateral propodeum with striations on ventral margin, around metapleural gland bulla, propodeal spiracle and posteroventral corner. All femora with hair-bearing punctures; punctures progressively denser apically on dorsal surface of profemur. All tibiae smooth and shining with dense punctulae and sparse macro hair-bearing punctures throughout. Petiole dorsum mostly smooth and shining with large hair-bearing punctures; lateral petiole surface smooth. Dorsum of first gastral tergite mostly smooth and shining with large, sparse, hair-bearing macro punctures; anterior face smooth and shining. Presternite IV imbricate. Abdominal segment IV with macro-punctures dorsally; sternite with very sparse macro punctures. Pygidium and hypopygium with sparse, hair-bearing macro punctures only.

***Pilosity.*** Mandibular dorsum with stout erect hairs directed inwards along basal margin, and short, decumbent hairs on dorsum. Anteriormost point clypeal margin with pair of long, stout, tubular peg-like setae and two decumbent hairs adjacent to the peg-like setae; clypeal dorsum with numerous, long erect hairs as well as decumbent short hairs sparsely distributed across surface. Head dorsum, between clypeus, antennal foramen, and eye with dense, semi-decumbent, appressed hairs; hairs posteriorly sparse with suberect hairs only; head dorsolaterally with decumbent hairs directed anteriorly. Scape with numerous subdecumbent and suberect hairs; flagella with numerous suberect and decumbent hairs. Mesosomal dorsum with erect and suberect hairs sparsely distributed. Suberect and decumbent hairs present on lateral surfaces of pronotum and propodeum. Coxae with many decumbent hairs reaching highest density on ventral surface. Femora with suberect hairs dorsally and subdecumbent hairs ventrally; tibiae with dense, decumbent hairs dorsally, and sparse decumbent hairs ventrally. All tarsi I with stout and apically blunt hairs arranged in a series, surrounded by simple decumbent hairs. Petiolar dorsum with decumbent hairs; lateral petiole surface with sparse decumbent hairs, almost glabrous; subpetiolar process with numerous suberect and erect hairs. Prora with numerous, fine, short, erect hairs directed anteriorly. All abdominal tergites and sternites with sparsely distributed suberect and erect hairs. Pygidium and hypopygium with numerous long erect hairs.

***Colour.*** Dark reddish brown across mesosoma, mandibles and legs distinctly paler red to orange in colour.

##### Ergatoid description.

With characters of worker but: lateral head margin not straight, distinctly widening posteriorly noticeably more so than the worker. Mesosoma stout and robust not as elongated as in worker; promesonotum distinctly convex; propodeum shorter, ~ 1/3 longer than propodeal declivity. Petiole nodiform, distinctly higher than long in lateral view; about as wide as long in dorsal view. Metasomal segments III-VII enlarged, segment III wider than petiole. Same colours as the worker.

##### Measurements.

Worker holotype: HL 1.13; HLL 0.87; HLA 0.23; HW 0.72; CML 0.22; SL 1.20; AII 0.21; AFI 0.32; AFII 0.24; EL 0.23; ML 0.56; PrL 0.71; PrH 0.49; PrW 0.66; WL 1.92; PeL 0.68; PeH 0.61; PeW 0.38; DPL 0.64; CI 63.57; CLI 19.8; SI 167.31; OI 26.67; LPI 89.66; DPI 59.75.

Worker paratypes (*n* = 12): HL 1.12–1.18; HLL 0.86–0.90; HLA 0.20–0.23; HW 0.69–0.75; CML 0.21–0.23; SL 1.16–1.26; AII 0.19–0.24; AFI 0.31–0.36; AFII 0.22–0.26; EL 0.22–0.25; ML 0.54–0.57; PrL 0.66–0.74; PrH 0.49–0.55; PrW 0.64–0.69; WL 1.79–1.99; PeL 0.60–0.69; PeH 0.56–0.61; PeW 0.33–0.39; DPL 0.52–0.67; CI 61.36–64.17; CLI 18.65–20.43; SI 166.25–172.94; OI 26.14–28.93; LPI 85.02–93.55; DPI 55.5–65.71.

Ergatoid paratype (*n* = 1): HL 1.19; HLL 0.90; HLA 0.259; HW 0.80; CML 0.25; SL 1.19; AII 0.23; AFI 0.30; AFII 0.22; EL 0.25; ML 0.59; PrL 0.73; PrH 0.56; PrW 0.7; WL 1.88; PeL 0.50; PeH 0.62; PeW 0.49; DPL 0.50; CI 67.31; CLI 21.34; SI 148.06; OI 28.28; LPI 123.01; DPI 97.61.

Non-type workers (*n* = 10): HL 1.12–1.18; HLL 0.82–0.92; HLA 0.16–0.24; HW 0.7–0.75; CML 0.2–0.25; SL 1.17–1.34; AII 0.18–0.22; AFI 0.31–0.36; AFII 0.23–0.26; EL 0.22–0.27; ML 0.55–0.61; PrL 0.66–0.74; PrH 0.5–0.55; PrW 0.64–0.71; WL 1.81–1.97; PeL 0.63–0.69; PeH 0.54–0.67; PeW 0.36–0.4; DPL 0.57–0.67; CI 61.82–64.78; CLI 18.01–20.73; SI 163.69–179.7; OI 25.74–31.04; LPI 81.83–97.24; DPI 56.97–68.64.

##### Morphological variation.

A spectrum of mesosomal size was observed, which is reflected in the variation seen within the mesosomal measurements. Moreover, the longitudinal ridges across the notopropodeal sulcus differs across the specimens examined with some showing more ribbing than others. No relationship between the size of a specimen and the longitudinal ridges could be found. Variation in both morphometric and qualitative characters mentioned above differs within the colony type series.

##### Comparative notes.

Within Xu and He (2015), specimens of *L.grohli* will key to the couplet containing both *L.peuqueti* and *L.confucii*. *Leptogenysgrohli* is distinguishable from *L.peuqueti* by the more linear lateral margins of the head than the convex lateral margins of *L.peuqueti*; the smaller eye (EL 0.22–0.27); and narrower head (HW 0.70–0.75). Sculptural differences also occur, with *L.grohli* having greater density of punctures across the whole head, scapes, mesosomal, petiole and first two gastral tergites than *L.peuqueti*. *Leptogenyspeuqueti* is generally larger in all aspects, with *L.grohli* being a smaller, more gracile species. *Leptogenysgrohli* is also morphologically similar to *L.confucii*, a species known from Taiwan and Southern Japan, but can be differentiated by the conspicuously defined angle between the basal and masticatory margin of the mandible; a smaller, less conspicuous frontal indentation; more angulated posterior head corner, and the lack of sculpture across the whole mesopleuron and lateral propodeal surfaces.

##### Distribution.

So far *L.grohli* is only known from Hong Kong and Guangdong This species, however, should be expected from neighbouring Chinese provinces and is likely to have been misidentified previously as *L.peuqueti*.

##### Ecology.

The type series was collected by sifting and extracting leaf litter within an old growth secondary forest. Specimens were noted during leaf litter sample collection, with the nest located within the soil between the tree trunk and its roots. One ergatoid and 16 workers were obtained, likely representing most of the colony. Solitary foraging individuals have been observed and collected during daytime across Hong Kong, with specimens obtained from tree plantations to secondary forests and Feng Shui Woods. Group hunting behaviour was not observed in this species.

##### Material examined.

***Holotype*** worker. China • 1 worker; Hong Kong SAR, Tai Po Kau Nature Reserve; 22.4271, 114.1814; 160 m a.s.l.; 23 Aug. 2022; A.I. Weemaels & M.T.Hamer leg.; Secondary forest, Winkler ex. leaf litter; ZRC ANTWEB1010093.

***Paratype*** workers: 12 workers: Same collection as holotype; ZRC ANTWEB1010085 to ANTWEB1010092. Same collection data as holotype; HKBM ANTWEB1010095 to ANTWEB1010099.

***Paratype*** ergatoid: Same collection data as holotype; ZRC ANTWEB1010094.

Non-type workers (*n* = 35): China • 3 workers; Hong Kong SAR, Tai Po Kau; 19 Jul. 1992; J.R.Fellowes leg.; HKBM MBS006896, MBS006897, MBS006899. • 2 workers; Guangdong Prov., Qi Mu Zhang; 6 Apr. 1997; J.R.Fellowes leg.; HKBM MBS015248. • 1 worker; Hong Kong SAR, Lung Fu Shan; 22.27876, 114.13728; 240 m a.s.l.; 24 Apr. 2015; R.H.Lee leg.; Plantation, Pitfall trap; IBBL RHL00050. • 2 worker; Hong Kong SAR, Lung Fu Shan; 22.2784, 114.1378; 230 m a.s.l.; 30 Apr. 2015; R.H.Lee leg.; Plantation, Pitfall trap; IBBL RHL00129, RHL00152. • 1 worker; Hong Kong SAR, Shing Mun; 22.39693, 114.153; 242 m a.s.l.; 14 May. 2015; R.H.Lee leg.; Feng Shui Forest, Pitfall trap; IBBL RHL00401. • 1 worker; Hong Kong SAR, Sunset Peak; 22.26112, 113.95633; 575 m a.s.l.; 3 Jun. 2015; R.H.Lee leg.; Secondary forest, Pitfall trap; IBBL RHL00892. • 1 worker; Hong Kong SAR, Sunset Peak; 22.26084, 113.95753; 575 m a.s.l.; 3 Jun. 2015; R.H.Lee leg.; Secondary forest, Pitfall trap; IBBL RHL00894. • 2 workers; Hong Kong SAR, Sunset Peak; 22.26392, 113.95376; 470 m a.s.l.; 3 Jun. 2015; R.H.Lee leg.; Secondary forest, Pitfall trap; IBBL RHL00906, RHL00908. • 1 worker; Hong Kong SAR, Sunset Peak; 22.26594, 113.95278; 440 m a.s.l.; 3 Jun. 2015; R.H.Lee leg.; Secondary forest, Pitfall trap; IBBL RHL00931. • 2 workers; Hong Kong SAR, Tai Po Kau; 22.42613, 114.18178; 160 m a.s.l.; 14 Jul. 2015; R.H.Lee leg.; Plantation, Pitfall trap; IBBL RHL00931, RHL02083. • 1 worker; Hong Kong SAR, Tai Po Kau; 22.42285, 114.18082; 200 m a.s.l.; 14 Jul. 2015; R.H.Lee leg.; Secondary forest, Pitfall trap; IBBL RHL02128. • 1 worker; Hong Kong SAR, Tai Po Kau; 22.42706, 114.17999; 180 m a.s.l.; 14 Jul. 2015; R.H.Lee leg.; Plantation, Pitfall trap; IBBL RHL02165. • 2 worker; Hong Kong SAR, Tai To Yan; 22.45479, 114.11821; 420 m a.s.l.; 4 Aug. 2015; R.H.Lee leg.; Secondary forest, Pitfall trap; IBBL RHL02211, RHL02686. • 3 worker; Hong Kong SAR, The Peak; 22.27523, 114.13873; 370 m a.s.l.; 17 Aug. 2015; R.H.Lee leg.; Secondary forest, Pitfall trap; IBBL RHL01090, RHL01099, RHL01106. • 1 worker; Hong Kong SAR, The Peak; 22.2767, 114.1423; 410 m a.s.l.; 17 Aug. 2015; R.H.Lee leg.; Secondary forest, Pitfall trap; IBBL RHL02621. • 2 worker; Hong Kong SAR, The Peak; 22.27603, 114.14199; 410 m a.s.l.; 17 Aug. 2015; R.H.Lee leg.; Secondary forest, Pitfall trap; IBBL RHL02636, RHL02639. • 1 worker; Hong Kong SAR, The Peak; 22.27495, 114.13828; 360 m a.s.l.; 17 Aug. 2015; R.H.Lee leg.; Secondary forest, Pitfall trap; IBBL RHL02655. • 1 worker; Hong Kong SAR, Tai Lam; 22.3956, 114.0928; 420 m a.s.l.; 26–29 Oct. 2015; R.H.Lee leg.; Bamboo forest, Pitfall trap; IBBL RHL02774. • 1 worker; Hong Kong SAR, Lung Fu Shan; 22.2758, 114.13546; 260 m a.s.l.; 30 Jun. 2016; R.H.Lee leg.; Secondary forest, General forager; IBBL RHL-SIA-007. • 1 worker; Hong Kong SAR, Mui Tsz Lam; 22.3892, 114.2345; 220 m a.s.l.; 4 Oct. 2016; R.H.Lee leg.; Secondary forest, Pitfall trap; IBBL RHL003304. • 1 worker; Hong Kong SAR, Tai Po Kau; 22.42613, 114.18178; 160 m a.s.l.; 6 Jun. 2017; R.H.Lee leg.; Secondary forest, Pitfall trap; IBBL RHL03549. • 1 worker; Hong Kong SAR, Tai Po Kau; 22.42706, 114.17999; 180 m a.s.l.; 6 Jun. 2017; R.H.Lee leg.; Secondary forest, Pitfall trap; IBBL RHL03555. • 1 worker; Hong Kong SAR, Lung Fu Shan; 22.2758, 114.13546; 250 m a.s.l.; 11 Oct. 2017; R.H.Lee leg.; Secondary forest, General forager; IBBL RHL-SIA-095. • 1 worker; Hong Kong SAR; Wong Lung Hang; 22.2658, 113.9528; 440 m a.s.l; 11 May. 2023; M.T.Hamer leg.; Shrubland, General-forager IBBL ANTWEB1010235.

##### Etymology.

Named after the musician Dave Grohl, lead singer, guitarist, and songwriter of the rock band Foo Fighters, drummer of the rock bands Nirvana and Queens of the Stone Age among others, for his positive activism, and ever long musical accompaniment to both first and last author.

#### ﻿ Leptogenys kitteli

Taxon classificationAnimaliaHymenopteraFormicidae

(Mayr, 1870)

653DFFE7-10BE-5157-9129-28E8BD521C91

**Figure 14.**
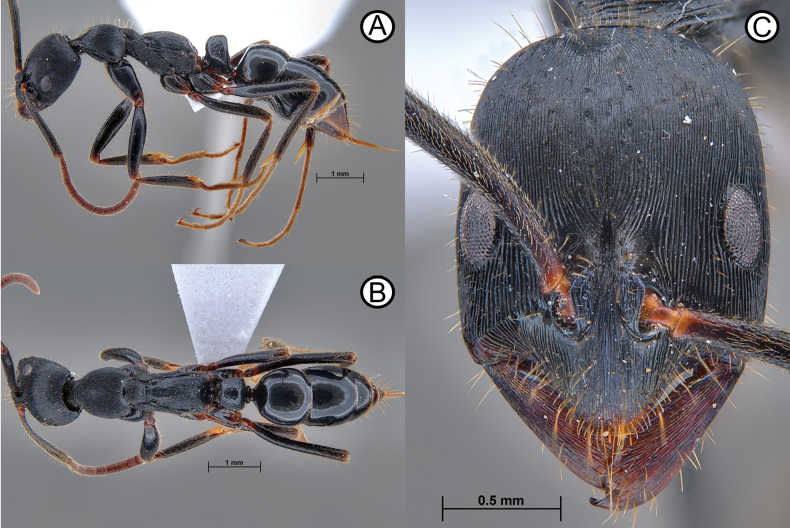
*Leptogenyskitteli* (PFL1T2W3-1) **A** lateral view **B** dorsal view **C** head in full face view.

Figs 7B , 8B , 14 , 20D

Lobopelta kitteli Mayr, 1870b: 966 (w.) INDIA (Sikkim).
Leptogenys kitteli : Emery 1895: 461.
Leptogenys kitteli altisquamis [senior synonym] Forel, 1900f: (w.) Myanmar: Xu and He 2015: 142. Of Leptogenyskitteliminor Forel, 1900f: 307 (w.) India: Xu and He 2015: 142. Of Leptogenyskittelisiemsseni Viehmeyer, 1922: 203, fig. 1 (w.) China: Xu and He 2015: 142.

##### Measurements.

Worker (*n* = 13): HL 1.72–1.87; HLL 1.37–1.53; HLA 0.37–0.47; HW 1.3–1.44; CML 0.33–0.45; SL 1.67–1.86; AII 0.21–0.32; AFI 0.32–0.37; AFII 0.29–0.33; EL 0.32–0.39; ML 0.73–0.94; PrL 0.97–1.14; PrH 0.7–0.85; PrW 0.97–1.06; WL 2.64–2.87; PeL 0.58–0.82; PeH 0.72–0.93; PeW 0.56–0.74; DPL 0.4–0.52; CI 71.27–81.04; CLI 18.21–25.62; SI 117.83–132; OI 21.94–26.5; LPI 107.98–158.96; DPI 116.12–147.26.

##### Morphological variation.

Specimens of *L.kitteli* show a wide range of variation in sculpture in different parts of the body. In most specimens, the fine costulae on the head run longitudinally across its entire surface; nonetheless, in some other specimens, some of the costulae converge medially, forming a concentric pattern similar to that found in *L.diminuta*. The mesosomal sculpturing varies even more when compared to the head sculpture; from fine costulae with interspaced foveae to irregular faint costulae throughout the mesosoma. The metanotal groove is deeply incised in the majority of specimens observed, with just a single specimen having a very shallow groove. Similar to *L.diminuta*, *L.kitteli* expresses a large degree of morphological variation across its wider geographical range and is likely a complex of species that would require further taxonomic investigation to resolve.

##### Comparative notes.

*Leptogenyskitteli* is most morphologically similar to *L.diminuta* within Hong Kong. Both species can be differentiated by the distinctly larger absolute size of *L.kitteli*, as well as the longitudinally striate pronotum, and absence of a longitudinal carinae on the clypeal dorsum.

##### Distribution.

*Leptogenyskitteli* is a widely distributed across the Indomalayan region, known from mainland China (Hainan, Guangxi, Yunnan, Sichuan, Guizhou, Hunan, Jiangxi, Fujian Zhejiang, Guangdong, Hubei, and Hong Kong SAR), Taiwan, India, Bangladesh, Myanmar, Thailand, Vietnam, Nepal (; Xu and He 2015; Bharti et al. 2016; Janicki et al. 2016; Guénard et al. 2017; Khachonpisitsak et al. 2020). This species should be expected from Laos and Cambodia.

##### Ecology.

*Leptogenyskitteli* is known to forage in large groups of workers, preying upon termites, as well as earthworms (Fellowes 1996). Similar to *L.diminuta*, colonies are known to move nesting locations and include up to several hundred individuals.

##### Material examined.

Workers (*n* = 36): China • 3 workers; Hong Kong SAR, Tai Mo Shan; 28 Sep. 1992; J.R.Fellowes leg.; HKBM MBS006891, MBS006892. • 1 worker; Hong Kong SAR, Victoria Park; 27 Jun. 1999; K. Eguchi leg.; HKBM MBS006576. • 1 worker; Hong Kong SAR, Victoria Park; 27 Jun. 1999; SK.Yamane leg.; SKYC MBS006586, ANTWEB1010189, ANTWEB1010190. • 3 workers; Guangdong Prov., Hei Shi Ding; 24 Apr. 1997; J.R.Fellowes leg.; HKBM MBS006906, MBS006907, MBS006908. • 1 worker; Guangdong Prov., Nankunshan; 20 Mar. 1997; J.R.Fellowes leg.; HKBM MBS015273. • 1 worker; Guangdong Prov., Qi Mu Zhang; 6 Apr. 1997; J.R.Fellowes leg.; HKBM MBS015271. • 2 workers; Guangdong Prov., Xin Jia Dong; 4 May. 1998; J.R.Fellowes leg.; HKBM MBS006577. • 2 workers; Hong Kong SAR, The Peak; 10 Sep. 2000; SK.Yamane leg.; SKYC ANTWEB1010186, ANTWEB1010187. • 1 worker; Hong Kong SAR, Aberdeen Reservoir; 22.26, 114.163; 160 m a.s.l.; 29 Jun. 2015; Yu-Ying Luo leg.; IBBL YYL00021. • 1 worker; Hong Kong SAR, Tai Tam; 22.2618, 114.2168; 360 m a.s.l.; 27 Jul. 2015; R.H.Lee leg.; Secondary forest, Pitfall trap; IBBL RHL02795. • 1 worker; Hong Kong SAR, The Peak; 22.27603, 114.14199; 400 m a.s.l.; 17 Aug. 2015; R.H.Lee leg.; Secondary Forest, Pitfall trap; IBBL RHL02638. • 1 worker; Hong Kong SAR, Tai Mo Shan; 22.41607, 114.12515; 800 m a.s.l.; 21 Jun. 2016; R.H.Lee leg.; Grassland, Pitfall trap; IBBL RHL03564. • 1 worker; Hong Kong SAR, Lung Fu Shan; 22.27603, 114.14199; 400 m a.s.l.; 11 Jul. 2016; R.H.Lee leg.; Secondary forest, Bait trap; IBBL RHL-SIA-091. • 1 worker; Hong Kong SAR, Southern Shek O; 22.25339, 114.24577; 190 m a.s.l.; 15 Jul. 2016; M.Pierce leg.; Secondary forest, Bait trap; IBBL ANTWEB1009151. • 1 worker; Hong Kong SAR, Deep Water Bay; 22.25483, 114.18338; 107 m a.s.l.; 12 Aug. 2016; M.Pierce leg.; Secondary forest, Hand collection; IBBL ANTWEB1009484. • 1 worker; Hong Kong SAR, Nam Fung Road; 22.2546, 114.1833; 120 m a.s.l.; 20 Aug. 2016; R.H.Lee leg.; Pitfall trap; IBBL RHL003365. • 1 worker; Hong Kong SAR, Deep Water Bay; 22.25483, 114.18338; 107 m a.s.l.; 12 Sep. 2016; M. Pierce leg.; Subtropical dry forest, ground forager; IBBL ANTWEB1009664. • 1 worker; Hong Kong SAR, Tai Mo Shan; 22.41099, 114.11917; 737 m a.s.l.; 2 Oct. 2016; C. Leung leg.; Secondary Forest, Bait trap; IBBL ANTWEB1009149. • 2 workers; Hong Kong SAR, Aberdeen; 22.25571, 114.16254; 99 m a.s.l.; 20–23 Oct. 2017; M.Pierce leg.; Secondary forest, Pitfall; IBBL ANTWEB1016994, ANTWEB1009645. • 1 worker; Hong Kong SAR, Tai Mo Shan; 22.4066, 114.12128; 760 m a.s.l.; 3 Oct. 2018; B. Morgan leg.; Hand collection; IBBL ANTWEB1010122. • 3 workers; Hong Kong SAR, Boa Vista; 22.2555, 114.2243; 260 m a.s.l.; 10 Jun. 2020; R.Wang leg.; Pitfall; IBBL RWB1236, RWB1237, RWB1238. • 2 workers; Hong Kong SAR, Pok Fu Lam; 22.26247, 114.1397; 217 m a.s.l.; 7 Apr. 2022; A. I. Weemaels & M.T.Hamer leg.; Secondary Forest, Winkler leaf litter Ex; IBBL PFL1T2W3-1, PFL1T2W5-1. • 6 workers; Hong Kong SAR; Magazine Gap; 22.26819, 114.16578; 255 m a.s.l.; 17 Oct. 2023; C.Y.L.Tse leg.; Young secondary forest, ex. nest; IBBL MTH680.

#### ﻿ Leptogenys kraepelini

Taxon classificationAnimaliaHymenopteraFormicidae

Forel, 1905

CD75E3D3-939B-51DB-A4E8-5EDEB72C0A28

**Figure 15.**
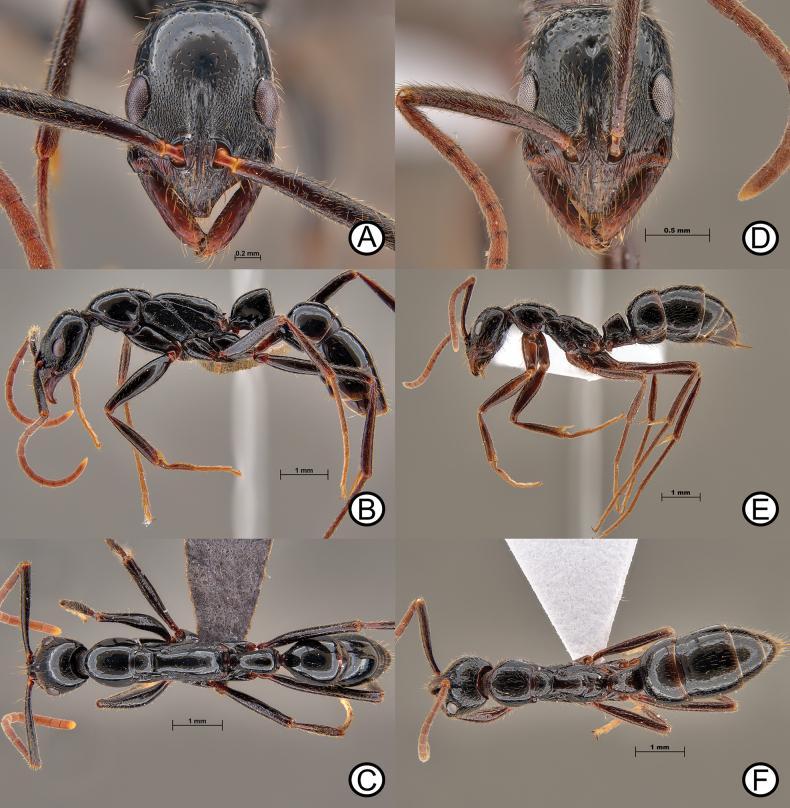
*Leptogenyskraepelini* (RHL00392) and ergatoid (ANTWEB1010172) **A** worker in lateral view **B** worker in dorsal view **C** worker in head in full face view **D** ergatoid in lateral view **E** ergatoid in lateral view and **F** ergatoid in dorsal view.

Figs 2B , 6A , 15 , 21A new record

Leptogenys (Lobopelta) kraepelini Forel, 1905f: 5 (w.) Indonesia (Java).
Leptogenys kraepelini baccha [senior synonym] Santschi, 1919: 336 (w.) Vietnam: Xu and He 2015: 142.

##### Measurements.

Workers (*n* = 15): HL 1.68–1.86; HLL 1.32–1.51; HLA 0.33–0.38; HW 1.09–1.23; CML 0.35–0.43; SL 1.7–2.04; AII 0.25–0.31; AFI 0.4–0.5; AFII 0.33–0.39; EL 0.41–0.47; ML 0.78–0.87; PrL 1.03–1.22; PrH 0.63–0.93; PrW 0.97–1.11; WL 2.91–3.26; PeL 0.92–1.07; PeH 0.87–0.99; PeW 0.35–0.65; DPL 0.9–1.02; CI 62.84–67.07; CLI 20.97–24.54; SI 153.66–174.8; OI 29.39–33.12; LPI 89.2–318.31; DPI 39.4–67.12.

Ergatoid queen (*n* = 1): HL 1.63; HLL 1.29; HLA 0.36; HW 1.67; CML 0.4; SL 1.53; AII 0.22; AFI 0.35; AFII 0.29; EL 0.42; ML 0.84; PrL 1.05; PrH 0.79; PrW 0.99; WL 2.71; PeL 0.73; PeH 0.84; PeW 0.55; DPL 0.71; CI 102.52; CLI 24.25; SI 91.8; OI 32.87; LPI 114.93; DPI 77.75.

##### Morphological variation.

Specimens collected in Hong Kong match well with the description provided by Forel, 1905 of *L.kraepelini*. However, the sculpture on the propodeum (as ‘sloping surface of the metanotum’ in Forel 1905) is not entirely ‘smooth’ (Forel 1905), but instead with transverse striations of varying degrees of pronunciation from few to many striae. Xu and He (2015) utilised this character (among others) to differentiate *L.kraepelini* from *L.chinensis* (Mayr, 1870), *L.chinensis* having transverse striation and *L.kraepelini* lacking it. Such striations appear absent in the dorsal image of the *L.kraepelini* (CASENT0281936, antweb.org) studied by Xu and He (2015). However, this specimen (from West Java) could represent a morphological extreme for this character. With a large distribution across Southeast Asia, this species likely shows a high degree of morphological variability similar to *L.diminuta* and *L.kitteli*. We suggest that this character should be treated with caution until the holotype specimen and additional material of *L.kraepelini* can be more closely scrutinised. Within Hong Kong, morphological variation appears to be limited. Most variation is associated with colour, with workers varying from reddish brown to jet-black, to black with a blue iridescent shine. The possibility of cryptic species within *L.kraepelini* and *L.chinensis* requires further taxonomic investigation which is not within the scope of this study. As such, despite ergatoids presented here we refrain from describing them.

##### Comparative notes.

This species highly resembles *L.peuqueti* and *L.grohli* within Hong Kong. However, *L.kraepelini* can be separated from both species by its much larger size, the distinctly posteriorly projecting propodeal lobes and the truncated anterior clypeal margin. *Leptogenyskraepelini* has been confused with *L.chinensis* in the past (Xu and He 2015). However, *L.kraepelini* has a rectangular head that does not significantly narrow behind the eyes as in *L.chinensis*; has punctuate sculpture between the clypeus and eyes which *L.chinensis* lacks, and a straight anterolateral clypeal margin which is sinuate in *L.chinensis*. More differing characters will likely be revealed upon closer inspection of type material and the examination of more specimens from across the wider region. Specimens previous determined to be *L.chinensis* from Guangdong were not examined in this study (Wu et al. 2008; Zhao et al. 2009). Such specimens should be re-examined, especially considering *L.kraepelini* is now confirmed from Guangdong and Hong Kong (this study), as well as the previously known misidentifications of *L.chinensis* for *L.kraepelini* (and *L.peuqueti*) in the past.

##### Distribution.

*Leptogenyskraepelini* is widely distributed across Indomalaya, recorded from China (Yunnan and Hong Kong SAR), Vietnam, Laos, Thailand, Malaysia Peninsula, Singapore, Borneo, Sumatra, and Java (Bakhtiar and Chiang 2010; Xu and He 2015; Janicki et al. 2016; Guénard et al. 2017; Khachonpisitsak et al. 2020; Wang et al. 2022). This species is currently absent from many southern Chinese provinces which likely reflects sampling effort in these regions, or has been incorrectly identified as *L.chinensis*.

##### Ecology.

This species is known to nest within rotting wood in young to old growth secondary and shrublands but is seemingly absent from highly disturbed environments. Specimens have been collected either individually or in small groups (three individuals) during the day, with colonies reproducing through ergatoid queens (Ito 1997). *Leptogenyskraepelini* appears to have a specialised diet on earwigs (Steghaus-Kovac and Maschwitz 1993) but has been observed preying upon termites in Hong Kong (CYLT pers. obs.).

##### Material examined.

Workers (*n* = 38): China • 1 worker; Hong Kong SAR, The Peak; 13 Jul. 1992; J.R.Fellowes leg.; HKBM MBS006905. • 1 worker; Hong Kong SAR, Hong Kong Island, The Peak; 24 Sep. 1993; J.R.Fellowes leg.; HKBM MBS015288. • 1 worker; Hong Kong SAR, The Peak; 27 Sep. 1994; J.R.Fellowes leg.; HKBM MBS006660. • 1 worker; Guangdong Prov. Daw U Ling; 28 Apr. 1997; J.R.Fellowes leg.; HKBM MBS015287 • 1 worker; Hong Kong SAR, Victoria Park; 27 Jun. 1999; SK.Yamane leg.; SKYC MBS006658, MBS006659. • 1 worker; Hong Kong SAR, Shing Mun Reservoir; 22.39737, 114.15268; 230 m a.s.l.; 6 Jul. 2011; P.S.Ward leg.; Secondary forest, ex. decay wood; IBBL ANTWEB1010182. • 1 worker; Hong Kong SAR, Shing Mun; 22.39693, 114.153; 240 m a.s.l.; 14 May. 2015; R.H.Lee leg.; Feng Shui Forest, Pitfall trap; IBBL RHL00364, RHL00367, RHL00392, RHL00407. • 1 worker; Hong Kong SAR, Sunset Peak; 22.266, 113.953; 430 m a.s.l.; 3 Jun. 2015; R.H.Lee leg.; Secondary forest, Pitfall trap; IBBL RHL00928. • 1 worker; Hong Kong SAR, Aberdeen Reservoir; 22.259, 114.16; 170 m a.s.l.; 26 Jun. 2015; R.H.Lee leg.; Secondary forest, Pitfall trap; IBBL RHL00861. • 1 worker; Hong Kong SAR, Aberdeen Reservoir; 22.262, 114.16; 180 m a.s.l.; 26 Jun. 2015; R.H.Lee leg.; Secondary forest, Pitfall trap; IBBL RHL01297. • 1 worker; Hong Kong SAR, The Peak; 22.277, 114.143; 450 m a.s.l.; 17 Aug. 2015; R.H.Lee leg.; Secondary forest, Pitfall trap; IBBL RHL02628. • 1 worker; Hong Kong SAR, Kap Lung; 22.41666, 114.10222; 256 m a.s.l.; 19 Sep. 2015; T.Tsang leg.; Winkler leaf litter Ex; IBBL TT01228. • 1 worker; Hong Kong SAR, Tai Tam; 22.396, 114.093; 420 m a.s.l.; 26 Oct. 2015; R.H.Lee leg.; Bamboo, Pitfall trap; IBBL RHL02778. • 1 worker; Hong Kong SAR, Shing Mun; 22.39693, 114.153; 240 m a.s.l.; 17 May. 2016; R.H.Lee leg.; Feng Shui Forest, Winkler leaf litter Ex; IBBL RHL03204. • 1 worker; Hong Kong SAR, Lung Fu Shan; 22.27603, 114.14199; 400 m a.s.l.; 11 Jul. 2016; R.H.Lee leg.; Secondary forest, Winkler leaf litter Ex; IBBL RHL-SIA-89. • 1 worker; Hong Kong SAR, Tai Lam Country Park; 22.37598, 114.04713; 200 m a.s.l.; 3 Nov. 2017; R.Cheung & M.Pierce leg.; Winkler leaf litter Ex; HKBM MBS011437. • 1 worker; Hong Kong SAR, Wong Lung Hang; 22.2668, 113.9524; 400 m a.s.l.; 11 May. 2023; M.T.Hamer leg.; Young secondary forest, Hand collection; IBBL ANTWEB101020. • 1 worker; Hong Kong SAR, Lantau; A Po Long; 22.28186, 113.9835; 200 m a.s.l.; 5 Jun. 2023; M.T.Hamer leg.; Young secondary forest, Hand collection; IBBL ANTWEB1010131, ANTWEB1010132, ANTWEB1010176. • 1 worker; Hong Kong SAR, Sunset Peak, Wong Lung Hang path; 22.2602, 113.95906; 650 m a.s.l.; 7 Jul. 2023; M.T.Hamer leg.; Montane forest, Hand collection; IBBL ANTWEB1010121. • 5 workers; Hong Kong SAR, Tai Mo Shan; 22.40585, 114.1063; 517 m a.s.l.; 26 Aug. 2023; M.T.Hamer leg.; Secondary forest, ex. Decay wood; IBBL MTH70, MTH157. • 1 worker; Hong Kong SAR, Tai Mo Shan; 22.40403, 114.10691; 471 m a.s.l.; 3 Sep. 2023; M.T.Hamer leg.; Secondary forest, Nest ex. decay log; IBBL ANTWEB1010169, ANTWEB1010174. • 1 worker; Hong Kong SAR, Tai Mo Shan; Kap Lung Forest Trail; 22.41088, 114.10451; 450 m a.s.l.; 3 Sep. 2023; M.T.Hamer leg.; Secondary forest, ex.decaying log; IBBL MTH276. • 1 worker; Hong Kong SAR, Severn Road; 22.27044, 114.15621; 380 m a.s.l.; 5 Sep. 2023; C.Y.L.Tse leg.; Nest ex.; IBBL ANTWEB1010168, ANTWEB1010175. • 4 workers; Hong Kong SAR, Wong Lung Hang; 22.26964, 113.95248; 265 m a.s.l.; 5 Sep. 2023; M.T.Hamer leg.; Secondary forest, ex .decaying log; IBBL MTH308, MTH323, MTH335, MTH336.

Ergatoids (*n* = 2): China • 1 ergatoid; Hong Kong SAR, Tai Mo Shan; Kap Lung Forest Trail; 22.41088, 114.10451; 450 m a.s.l.; 3 Sep. 2023; M.T.Hamer leg.; Secondary forest, nest ex. decaying log; IBBL MTH276. • 1 ergatoid; Hong Kong SAR, Severn Road; 22.27044, 114.15621; 380 m a.s.l.; 5 Sep. 2023; C.Y.L.Tse leg.;, Nest ex.; IBBL ANTWEB1010172.

#### ﻿ Leptogenys laeviterga

Taxon classificationAnimaliaHymenopteraFormicidae

Zhou et al., 2012

1E3647BD-F821-5880-A91C-3D16B3BE9B51

**Figure 16.**
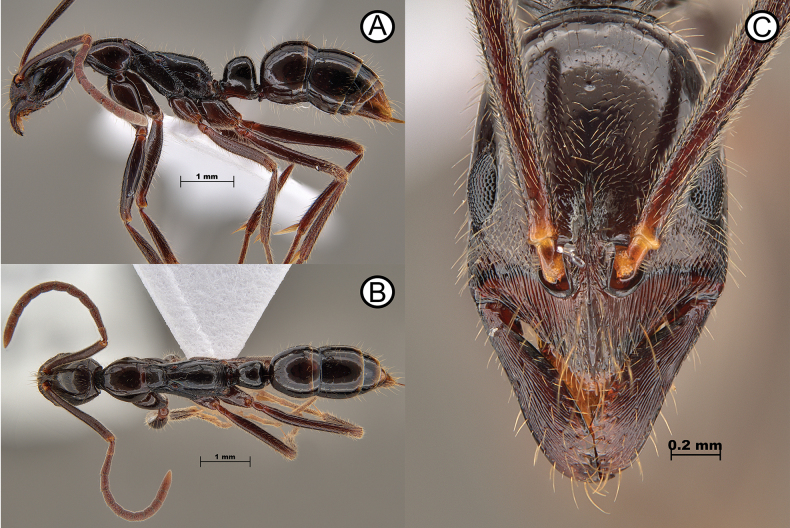
*Leptogenyslaeviterga* (ANTWEB1010142) **A** lateral view **B** dorsal view **C** head face view.

Figs 7A , 16 , 21B new record

Leptogenys laeviterga Zhou et al., 2012: 888, figs 1–3 (w.) China (Guangxi).

##### Measurements.

Worker (*n* = 1): HL 1.58; HLL 1.22; HLA 0.31; HW 1.07; CML 0.4; SL 1.99; AII 0.28; AFI 0.43; AFII 0.34; EL 0.28; ML 0.92; PrL 1.03; PrH 0.65; PrW 0.93; WL 2.78; PeL 0.76; PeH 0.84; PeW 0.59; DPL 0.62; CI 67.76; CLI 25.35; SI 185.26; OI 22.89; LPI 110.58; DPI 94.2.

##### Morphological variation.

Owing to the poor quality and quantity of specimens collected, little is known about the morphological variability of this species.

##### Comparative notes.

*Leptogenyslaeviterga* is superficially similar to *L.diminuta* owing to similarly shaped triangular mandibles, broad petiole shape, as well as overall body size. However, *L.laeviterga* is readily differentiated by the distinct and conspicuous median clypeal carina; lack of costulate sculpture on the head, lack of teeth on the mandibular masticatory margin, and longer scapes (SL 1.90–1.99). Within the wider Indomalayan *Leptogenys* fauna, *L.laeviterga* is morphologically similar to *L.sunzii* Xu & He, 2015, but can be differentiated by the truncated clypeal apex in *L.laeviterga* (pointed and convex in *L.sunzii*), the smaller eyes (larger in *L.sunzii*), and the higher than long petiole in *L.sunzii* whereas the petiole as long as high in *L.laeviterga* (Xu and He 2015).

##### Distribution.

Previously, *L.laeviterga* was only known from its type locality in Darning Mountain National Nature Reserve, Guangxi (Zhou et al. 2012). Here we provide the first record of the species from Hong Kong, representing the eastern most record for this species thus far. The species should therefore be expected from Guangdong and other neighbouring provinces in China.

##### Ecology.

Very little is known of the ecology of *L.laeviterga*. Specimens (but not the whole colony) from Hong Kong were obtained from within a decaying log from an old growth secondary forest on the southern slopes of Tai Mo Shan (471 m a.s.l.), Hong Kong. Male specimens were obtained on the day of collection. Considering the sampling effort undertaken in Hong Kong, it is surprising that more *L.laeviterga* have not been collected, indicating the potential rarity of this species.

##### Material examined.

Workers (*n* = 3): China • 3 workers; Hong Kong SAR, Tai Mo Shan; 22.40403, 114.10691; 471 m a.s.l.; 26 Aug. 2023; M.T.Hamer leg.; Secondary forest, ex. decay log; IBBL ANTWEB1010142, ANTWEB1010155, ANTWEB1010156.

#### ﻿ Leptogenys peuqueti

Taxon classificationAnimaliaHymenopteraFormicidae

(André, 1887)

E0049570-5DA0-5E1F-892A-7F1DD7A165B2

**Figure 17.**
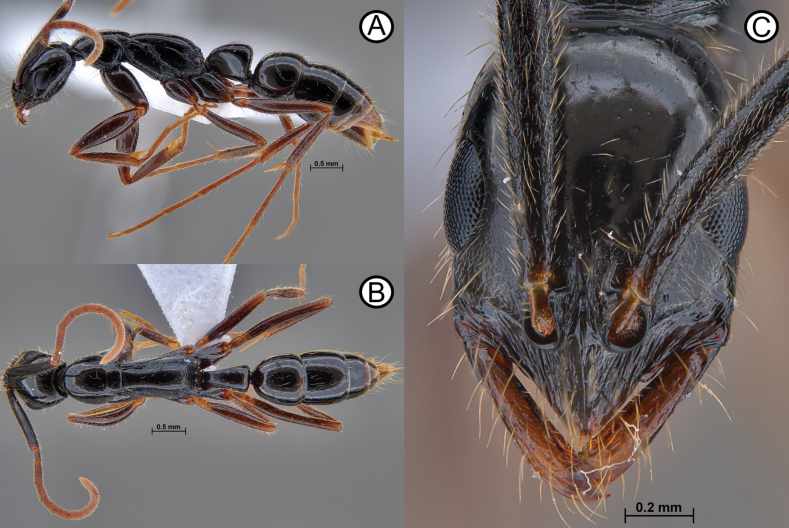
*Leptogenyspeuqueti* (FK1T2W5-1) **A** lateral view **B** dorsal view **C** head in full face view.

Figs 3B, D , 17 , 21C

Lobopelta peuqueti André, 1887: 292 (w.) Vietnam.
Leptogenys : Emery, 1895m: 461.
Leptogenys minchinii [senior synonym] Forel, 1900f: 308 (w.) India, Myanmar: Xu and He 2015: 145. Of Leptogenyspeuquetiwatsoni Forel, 1900f: 309 (w.) Myanmar: Xu and He 2015: 145.

##### Measurements.

Worker (*n* = 15): HL 1.15–1.31; HLL 0.88–1.02; HLA 0.17–0.28; HW 0.75–0.87; CML 0.23–0.29; SL 1.19–1.39; AII 0.17–0.21; AFI 0.34–0.39; AFII 0.25–0.3; EL 0.29–0.37; ML 0.5–0.65; PrL 0.73–0.85; PrH 0.51–0.74; PrW 0.6–0.76; WL 2.02–2.2; PeL 0.67–0.75; PeH 0.52–0.63; PeW 0.33–0.45; DPL 0.66–0.75; CI 64.63–69.92; CLI 19.91–22.41; SI 156.24–167.09; OI 30.69–37.14; LPI 74.93–90.7; DPI 47.94–65.32.

##### Morphological variation.

Morphological variation with *L.peuqueti* specimens collected from Hong Kong consists of overall size variation, with some specimens being larger and more robust than others collected. Such variation can be seen with the dorsal pronotal width and Webers length (PrW 0.6–0.76; WL 2.02–2.2). Additional variation is associated with colour, with workers varying from jet-black, to black with an iridescent blue shine.

##### Comparative notes.

*Leptogenyspeuqueti* is most similar to *L.grohli* and *L.kraepelini* within Hong Kong and Macao, being distinguishable from *L.grohli* by the smooth head dorsum, larger eyes, shorter tubular setae on the anterior clypeal margin, and convex lateral head margins. *Leptogenyspeuqueti* highly resembles *L.kraepelini*, but lacks the truncated anterior clypeal margin found in *L.kraepelini*, the posteriorly projecting propodeal lobes and is overall smaller. Within the wider Indomalayan *Leptogenys* species, *L.peuqueti* is most similar to *L.confucii*, as well as other members of the *L.chinensis* group. *Leptogenyspeuqueti* can be differentiated by the smooth head dorsum, meso- and metathorax, and propodeum, as well as its black colour.

##### Distribution.

A very wide-ranging species in Indomalaya, *L.peuqueti* is recorded from several Chinese provinces including Fujian, Guangdong, Guangxi, Hainan, Hong Kong Hubei, Hunan, Macao, Yunnan, and Zhejiang (Guénard and Dunn 2012; Xu and He 2015; Janicki et al. 2016; Guénard et al. 2017; Brassard et al. 2021). Other countries include Bangladesh, Bhutan, India (Andaman Islands, Kerala, Meghalaya, Sikkim, and West Bengal), Indonesia (Java), Malaysia (Peninsular and Bornean parts), Myanmar, The Philippines, Singapore, Sri Lanka, and Vietnam (Bakhtiar and Chiang 2010; Janicki et al. 2016; Guénard et al. 2017; Wang et al. 2022). Further sampling effort across the Oriental region will likely produce new country level records of this species (e.g., Cambodia, Laos, Thailand).

##### Ecology.

*Leptogenyspeuqueti* is the most common *Leptogenys* recorded from Hong Kong, collected from a wide variety of habitats including forests, shrubland as well as disturbed urban sites. Workers are known to foraging individually but will recruit small numbers of workers to tackle larger prey items (Janssen et al. 1997; MTH pers. obs.). Workers have been noted to feed upon isopods in Hong Kong. Nests have been found within rotting logs, under rocks and within soil (often underneath objects). Colonies are queenless, instead reproducing through gamergates (Ito 1997). One colony collection contained 67 workers, 20 cocoons and four larvae (MTH271), correlating with Ito (1997) who found the number of workers in *L.peuqueti* colonies to range from 5 and 97.

##### Material examined.

Workers (*n* = 134): China • 1 worker; Hong Kong SAR, Sunset Peak; 25 Jul. 1992; J.R.Fellowes leg.; HKBM MBS06893. • 1 worker; Hong Kong SAR, Lion Rock; 27 Aug. 1992; J.R.Fellowes leg.; HKBM MBS006901. • 1 worker; Hong Kong SAR, Lion Rock; 27 Aug. 1992; J.R.Fellowes leg.; HKBM MBS006902. • 1 worker; Hong Kong SAR, Lion Rock; 27 Aug. 1992; J.R.Fellowes leg.; HKBM MBS006903. • 1 worker; Guangdong Prov., Ding Hu Shan; 25 Sep. 1995; J.R.Fellowes leg.; HKBM MBS015247. • 1 worker; Hong Kong SAR, Luk Tei Tong, Lantau; 7 Oct. 1996; J.R.Fellowes leg.; IBBL MBS006606. • 1 worker; Hong Kong SAR, Lantau, Tong Fuk; 4 Nov. 1996; J.R.Fellowes leg.; HKBM MBS015246. • 1 worker; Hong Kong SAR, Tong Fuk, Lantau Island; 4 Nov. 1996; J.R.Fellowes leg.; IBBL MBS006889. • 1 worker; Hong Kong SAR, Tong Fuk, Lantau Island; 4 Nov. 1996; J.R.Fellowes leg.; HKBM MBS006890. • 1 worker; Hong Kong SAR, Lantau, Tei Tong Tsai; 5 Nov. 1996; J.R.Fellowes leg.; HKBM MBS015261. • 1 worker; Hong Kong SAR, Nam Long, Western New Territories; 7 Nov. 1996; J.R.Fellowes leg.; HKBM MBS015255. • 1 worker; Hong Kong SAR, Kadoorie Farm Botanical Garden; 460 m a.s.l.; 21 Sep. 1999; J.R.Fellowes leg.; HKBM MBS015254. • 1 worker; Hong Kong SAR, The Peak; 10 Sep. 2000; SK.Yamane leg.; SKYC ANTWEB1010194. • 1 worker; Hong Kong SAR, Castle Peak; 22.391, 113.958; 230 m a.s.l.; 30 Jun. 2015; R.H.Lee leg.; Shrubland, Pitfall trap; IBBL RHL01178. • 1 worker; Hong Kong SAR, Castle Peak; 22.391, 113.958; 230 m a.s.l.; 30 Jun. 2015; R.H.Lee leg.; Shrubland, Pitfall trap; IBBL RHL01200. • 1 worker; Hong Kong SAR, Discovery Bay; 22.31, 114.018; 5 m a.s.l.; 2 Jul. 2015; R.H.Lee leg.; Grassland, Pitfall trap; IBBL RHL01907. • 1 worker; Hong Kong SAR, Tai Tam; 22.262, 114.217; 370 m a.s.l.; 27 Jul. 2015; R.H.Lee leg.; Secondary forest, Pitfall trap; IBBL RHL02792. • 2 worker; Hong Kong SAR, Tap Mun; 22.481, 114.361; 100 m a.s.l.; 28 Jul. 2015; R.H.Lee leg.; Shrubland, Pitfall trap; IBBL RHL01540, RHL01547. • 1 worker; Hong Kong SAR, Tap Mun; 22.477, 114.363; 45 m a.s.l.; 28 Jul. 2015; R.H.Lee leg.; Shrubland, Pitfall trap; IBBL RHL01605, RHL01619. • 1 worker; Hong Kong SAR, Chap Lap Kok; 22.29301, 113.93407; 50 m a.s.l.; 17 Sep. 2015; B.Morgan leg.; Winkler leaf litter Ex; IBBL BMW00181. • 1 worker; Hong Kong SAR, Chap Lap Kok; 22.29301, 113.93407; 50 m a.s.l.; 27 Sep. 2015; B.Morgan leg.; Hand collection; IBBL BMW00247, BMW00248. • 1 worker; Hong Kong SAR, Nam Fung Road; 22.25553, 114.18015; 108 m a.s.l.; 1 Oct. 2015; T.Tsang leg.; Winkler leaf litter Ex; IBBL TT01495. • 1 worker; Hong Kong SAR, Chap Lap Kok; 22.29301, 113.93407; 50 m a.s.l.; 12 Oct. 2015; B.Morgan leg.; Winkler leaf litter Ex; IBBL BMW01086. • 1 worker; Hong Kong SAR, Sha Shan; 22.449, 114.145; 50 m a.s.l.; 3 Nov. 2015; R.H.Lee leg.; Feng Shui Forest, Pitfall trap; IBBL RHL02755. • 1 worker; Hong Kong SAR, Chap Lap Kok; 22.2947, 113.9336; 20 m a.s.l.; 19 Mar. 2016; B.Morgan leg.; Winkler leaf litter Ex; IBBL BMW00715. • 1 worker; Hong Kong SAR, Chap Lap Kok; 22.2929, 113.9347; 60 m a.s.l.; 5 Apr. 2016; B.Morgan leg.; Winkler leaf litter Ex; IBBL BMW02365. • 1 worker; Hong Kong SAR, Chap Lap Kok; 22.2918, 113.9333; 30 m a.s.l.; 19 Apr. 2016; B.Morgan leg.; Hand collection; IBBL BMW00581. • 4 worker; Hong Kong SAR, Nam Fung Road; 22.2546, 114.1833; 120 m a.s.l.; 20 Aug. 2016; R.H.Lee leg.; Feng Shui Forest, Pitfall trap; IBBL RHL03518, RHL03360, RHL003381, RHL03391. • 1 worker; Hong Kong SAR, Southern Aberdeen; 22.25109, 114.16259; 99 m a.s.l.; 19 Sep. 2016; M.Pierce leg.; subtropical dry forest, Hand collection; IBBL ANTWEB1009470. • 1 worker; Hong Kong SAR, Tai Po: Kadoorie; 22.43332, 114.1172; 191 m a.s.l.; 21 Sep. 2016; M.Pierce leg.; botanical garden, Hand collection; IBBL ANTWEB1009083. • 2 worker; Hong Kong SAR, Pak Ngan Heung; 22.271, 113.9891; 35 m a.s.l.; 25 Oct. 2016; R.H.Lee leg.; Feng Shui Forest, Pitfall trap; IBBL RHL003347, RHL03508. • 1 worker; Hong Kong SAR, Pak Ngan Heung; 22.27099, 113.98911; 35 m a.s.l.; 25 Oct. 2016; R.H.Lee leg.; Feng Shui Forest, Pitfall trap; IBBL RHL03509. • 1 worker; Hong Kong SAR, Mai Po; 22.487, 114.0392; 1 m a.s.l.; 26–20 Oct. 2016; R.H.Lee leg.; Wetlands, Pitfall trap; IBBL RHL002943. • 1 worker; Hong Kong SAR, Chap Lap Kok; 22.2947, 113.9336; 20 m a.s.l.; 27 Nov. 2016; B.Morgan leg.; Hand collection; IBBL BMW02465. • 1 worker; Hong Kong SAR, Soko Island; 22.18258, 113.91257; 3 m a.s.l.; 2 Dec. 2016; M.Pierce leg.; Forest, Hand collection; IBBL ANTWEB1009836. • 1 worker; Hong Kong SAR, Chap Lap Kok; 22.2947, 113.9336; 30 m a.s.l.; 28 Feb. 2017; B.Morgan leg.; Winkler leaf litter Ex; IBBL BMW02022. • 1 worker; Hong Kong SAR, Lok Ma Chau; 22.51023, 114.06352; 1 m a.s.l.; 9 Apr. 2017; M.Pierce leg.; Pitfall; IBBL ANTWEB1016941, ANTWEB1017005. • 1 worker; Hong Kong SAR, Lok Ma Chau; 22.51044, 114.06348; 21 m a.s.l.; 9–12 May.2017; M.Pierce leg.; Pitfall; IBBL ANTWEB1016958. • 2 worker; Hong Kong SAR, Lok Ma Chau; 22.51117, 114.06576; 6 m a.s.l.; 11–14 Aug. 2017; M.Pierce leg.; Wetland, Pitfall; IBBL ANTWEB1017019, ANTWEB1009760. • 1 worker; Hong Kong SAR, Lion Rock; 22.357, 114.17504; 140 m a.s.l.; 15 Aug. 2017; R.H.Lee leg.; Secondary forest, Pitfall trap; IBBL RHL03526. • 1 worker; Hong Kong SAR, Pak Tam Chung; 22.39474, 114.32314; 70 m a.s.l.; 6 Sep. 2017; R.H.Lee leg.; Shrubland, Pitfall trap; IBBL RHL03492. • 3 worker; Hong Kong SAR, Mai Po; 22.4849, 114.03298; 3 m a.s.l.; 12–15 Sep. 2017; M.Pierce leg.; Pitfall; IBBL ANTWEB1009693, ANTWEB1016943, ANTWEB1009657. • 2 worker; Hong Kong SAR, Mai Po; 22.4849, 114.03298; 3 m a.s.l.; 12–15 Sep. 2017; M.Pierce leg.; Pitfall; IBBL ANTWEB1009657, ANTWEB1009713. • 1 worker; Hong Kong SAR, Mai Po; 22.49413, 114.04014; 3 m a.s.l.; 26–29 Sep.2017; M.Pierce leg.; Pitfall; IBBL ANTWEB1009710. • 4 workers; Hong Kong SAR, Mai Po; 22.49473, 114.03919; 3 m a.s.l.; 26–29 Sep.2017; M.Pierce leg.; Pitfall; IBBL ANTWEB1009614, ANTWEB1009688, ANTWEB1009616, ANTWEB1009783. • 1 worker; Hong Kong SAR, Mai Po; 22.49413, 114.04014; 3 m a.s.l.; 26–29 Sep.2017; M.Pierce leg.; Pitfall; IBBL ANTWEB1009782. • 2 worker; Hong Kong SAR, Tung Ping Chau; 22.5382, 114.4365; 30 m a.s.l.; 2 Oct. 2017; R.Cheung & B.Morgan leg.; Winkler leaf litter Ex; HKBM MBS011433, MBS011434. • 1 worker; Hong Kong SAR, Tung Ping Chau; 22.5443, 114.4317; 1 m a.s.l.; 2 Oct. 2017; R.Cheung & B.Morgan leg.; Winkler leaf litter Ex; HKBM MBS011435. • 2 worker; Hong Kong SAR, Penny’s Bay; 22.32588, 114.03384; 22 m a.s.l.; 10–13 Oct.2017; M.Pierce leg.; Pitfall; IBBL ANTWEB1009781, ANTWEB1017072. • 1 worker; Hong Kong SAR, Kam Shan Country Park; 22.3562, 114.15167; 170 m a.s.l.; 18 Oct. 2017; R.Cheung leg.; Winkler leaf litter Ex; HKBM MBS011436. • 2 worker; Hong Kong SAR, Penny’s Bay; 22.32719, 114.0336; 21 m a.s.l.; 23–26 Oct.2017; M.Pierce leg.; Pitfall; IBBL ANTWEB1016974, ANTWEB1017001. • 2 worker; Hong Kong SAR, Lung Fu Shan; 22.279, 114.1361; 242 m a.s.l.; 20 Jun. 2019; K.Chan leg.; IBBL ANTWEB1010118, ANTWEB1010119. • 3 worker; Hong Kong SAR, Luk Keng Chan Uk; 22.5183, 114.2187; 10 m a.s.l.; 5 Aug. 2020; R.Wang leg.; IBBL RWB1239, RWB1240, RWB1241. • 1 worker; Hong Kong SAR, Mai Po; 22.47912, 114.03847; 5 m a.s.l.; 18 Aug. 2021; T.Bogar leg.; Grassland, Pitfall; IBBL ANTWEB1010207. • 1 worker; Hong Kong SAR, Mai Po; 22.4801, 114.03814; 5 m a.s.l.; 18 Aug. 2021; T.Bogar leg.; Grassland, Pitfall; IBBL ANTWEB1010208. • 2 worker; Hong Kong SAR, Lok Ma Chau; 22.51093, 114.0652; 5 m a.s.l.; 2 Sep. 2021; T.Bogar leg.; Grassland, Pitfall; IBBL ANTWEB1010210. • 1 worker; Hong Kong SAR, Lok Ma Chau; 22.51106, 114.0656; 5 m a.s.l.; 2 Sep. 2021; T.Bogar leg.; Grassland, Pitfall; IBBL ANTWEB1010211. • 3 worker; Hong Kong SAR, Lok Ma Chau; 22.51093, 114.0652; 5 m a.s.l.; 2 Sep. 2021; T.Bogar leg.; Grassland, Pitfall; IBBL ANTWEB1010212, ANTWEB1010214, ANTWEB1010219. • 1 worker; Hong Kong SAR, Lok Ma Chau; 22.51106, 114.0656; 5 m a.s.l.; 2 Sep. 2021; T.Bogar leg.; Grassland, Pitfall; IBBL ANTWEB1010221. • 1 worker; Hong Kong SAR, Wetlands Park; 22.47236, 114.00401; 1 m a.s.l.; 7 Sep. 2021; A. I. Weemaels & M.T.Hamer leg.; Wetlands, Winkler leaf litter Ex; IBBL WP1T2W2-7. • 1 worker; Hong Kong SAR, Mai Po; 22.47957, 114.03742; 5 m a.s.l.; 14 Sep. 2021; T.Bogar leg.; Grassland, Pitfall; IBBL ANTWEB1010206. • 1 worker; Hong Kong SAR, Lok Ma Chau; 22.51076, 114.06466; 5 m a.s.l.; 16 Sep. 2021; T.Bogar leg.; Grassland, Pitfall; IBBL ANTWEB1010217. • 1 worker; Hong Kong SAR, Lok Ma Chau; 22.51076, 114.06466; 5 m a.s.l.; 16 Sep. 2021; T.Bogar leg.; Grassland, Pitfall; IBBL ANTWEB1010218. • 1 worker; Hong Kong SAR, Lok Ma Chau; 22.51057, 114.06412; 5 m a.s.l.; 16 Sep. 2021; T.Bogar leg.; Grassland, Pitfall; IBBL ANTWEB1010220. • 2 worker; Hong Kong SAR, Lok Ma Chau; 22.51038, 114.06371; 5 m a.s.l.; 28 Sep. 2021; T.Bogar leg.; Grassland, Pitfall; IBBL ANTWEB1010213, ANTWEB1010215. • 1 worker; Hong Kong SAR, Lok Ma Chau; 22.51176, 114.06592; 5 m a.s.l.; 29 Sep. 2021; T.Bogar leg.; Grassland, Pitfall; IBBL ANTWEB1010216. • 1 worker; Hong Kong SAR, Mai Po; 22.48365, 114.03868; 5 m a.s.l.; 2 Oct. 2021; T.Bogar leg.; Grassland, Pitfall; IBBL ANTWEB1010209. • 1 worker; Hong Kong SAR, Cheng Chau Island; 22.19814, 114.0199; 20 m a.s.l.; 25 Nov. 2021; M.T.Hamer leg.; Garden, ex. Decay wood; IBBL MTH635. • 1 worker; Hong Kong SAR, Fung Kong; 22.51322, 114.0937; 30 m a.s.l.; 18 May. 2022; A. I. Weemaels leg.; Young secondary forest, Winkler leaf litter Ex; IBBL FK1T2W5-1. • 2 workers; Hong Kong SAR, Fanling Golf Course; 22.49009, 114.10847; 54 m a.s.l.; 23–25 May. 2022; A. I. Weemaels & M.T.Hamer leg.; Old secondary forest, Pitfall trap; IBBL FGE1SQ2PF4-1. • 1 worker; Hong Kong SAR, Wai Tsai; 22.48284, 114.06153; 29 m a.s.l.; 29 Jun. 2022; M.T.Hamer leg.; Vacuum; IBBL WT2SQ1GVAC3-1. • 1 worker; Hong Kong SAR, Fung Kong; 22.5322, 114.0937; 47 m a.s.l.; 25 Jul. 2022; A. I. Weemaels & M.T.Hamer leg.; Young secondary, Winkler leaf litter Ex; IBBL FK3GC7-17. • 1 worker; Hong Kong SAR, Tai Po Kau Headland; 22.43841, 114.19261; 45 m a.s.l.; 18 Aug. 2022; M.T.Hamer leg.; Old secondary forest, Log Sift; IBBL TPK4GC16-1. • 1 worker; Hong Kong SAR, Tai Po Kau Headland; 22.43471, 114.19264; 52 m a.s.l.; 18 Aug. 2022; M.T.Hamer leg.; Old secondary forest, Log Sift; IBBL TPK4GC17-4. • 2 worker; Hong Kong SAR, Tai Po Kau Headland; 22.43841, 114.19261; 45 m a.s.l.; 18–24 Aug.2022; M.T.Hamer & T.S.R.Silva leg.; Old secondary forest, Pitfall trap; IBBL TPK4SQ1PF4-8, TPK4SQPF1-9. • 4 workers; Hong Kong SAR, Tai Mo Shan; 22.40346, 114.1068; 464 m a.s.l.; 26 Aug. 2023; M.T.Hamer leg.; Secondary forest, ex. Decay wood; IBBL MTH159. • 5 workers; Hong Kong SAR, Lantau; 22.25157, 113.9407; 273 m a.s.l.; 28 Aug. 2023; M.T.Hamer leg.; Young secondary, ex. Decaying log; IBBL MTH214. • 1 worker; Hong Kong SAR, Lantau; 22.2516, 113.94070; 273 m a.s.l; 28 Aug. 2023; M.T.Hamer leg.; Young secondary forest, ex. Nest; IBBL MTH192. • 5 workers; Hong Kong SAR, Shui Hau; 22.2149, 113.91580 56 m a.s.l; 31 Aug. 2023; M.T.Hamer leg.; un. Log & ex. Soil, Young secondary forest IBBL MTH271. • 5 worker; Hong Kong SAR, Tai Mo Shan; 22.40403, 114.10691; 471 m a.s.l.; 03 Sep. 2023; M.T.Hamer leg.; secondary forest, ex. Decaying log; IBBL MTH275. • 1 worker; Hong Kong SAR, Guilford Road; 22.2649, 114.16690; 380 m a.s.l.; 05 Sep. 2023; C.Y.L.Tse leg.; urban green space, ex. Nest; IBBL MTH363. • 5 workers; Hong Kong SAR, Fanling Golf Course; 22.4915, 114.11977; 40 m a.s.l.; 27 Sep. 2023; M.T.Hamer leg.; Old secondary forest, un. Log & ex. Soil; IBBL MTH621.

#### ﻿ Leptogenys rufida

Taxon classificationAnimaliaHymenopteraFormicidae

Zhou et al., 2012

C57A66AA-3101-5903-81BF-545D3DE9F4DD

**Figure 18.**
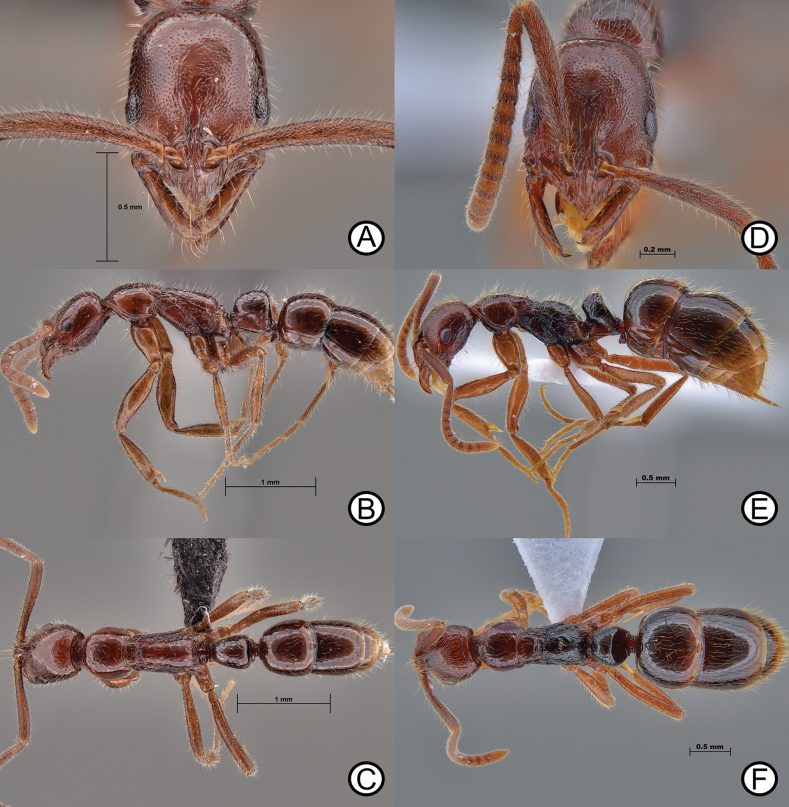
*Leptogenysrufida* (RHL01259) and ergatoid (ANTWEB1010234) **A** worker in lateral view **B** worker in dorsal view **C** worker in head in full face view **D** ergatoid in lateral view **E** ergatoid in lateral view and **F** ergatoid in dorsal view.

Figs 2A , 3A, C , 18 , 21D new record

Leptogenys rufida Zhou et al., 2012: 891, figs 4–6 (w.) China (Guangxi).

##### Ergatoid description.

With characters of worker but head more square, not as long as in worker. Clypeus terminating in less convex point apically. Propodeum shorter; convex in lateral view. Petiole nodiform, distinctly higher than long in lateral view; wider than long in dorsal view; anterior margin straight in lateral view. Metasomal segments III–VII enlarged, segment III distinctly wider than petiole. Same colour as the worker (Zhou et al. 2012).

##### Measurements.

Workers (*n* = 8): HL 0.93–1.01; HLL 0.76–0.8; HLA 0.18–0.21; HW 0.64–0.67; CML 0.19–0.21; SL 0.84–0.92; AII 0.17–0.19; AFI 0.13–0.15; AFII 0.12–0.14; EL 0.16–0.18; ML 0.45–0.46; PrL 0.53–0.6; PrH 0.38–0.44; PrW 0.56–0.59; WL 1.38–1.49; PeL 0.46–0.51; PeH 0.42–0.56; PeW 0.35–0.42; DPL 0.42–0.48; CI 64.29–68.17; CLI 19.36–22.51; SI 129.66–141.82; OI 20.78–23.37; LPI 82.61–114.29; DPI 76.76–90.83.

Ergatoid paratype (*n* = 1): HL 1.04; HLL 0.78; HLA 0.21; HW 0.74; CML 0.25; SL 0.89; AII 0.18; AFI 0.14; AFII 0.13; EL 0.19; ML 0.52; PrL 0.66; PrH 0.45; PrW 0.61; WL 1.5; PeL 0.43; PeH 0.61; PeW 0.5; DPL 0.25; CI 71.22; CLI 23.77; SI 120.41; OI 24.55; LPI 141.92; DPI 202.01.

##### Morphology.

Little to no variation is detected with the specimens examined, other than subtle differences in the development of the longitudinal ribbing across the mesonotal notch, however in no specimens was this character absent.

##### Comparative notes.

*Leptogenysrufida* is the smallest *Leptogenys* known from Hong Kong (WL 1.38–1.48) and is further recognisable by the following combined morphological characters; subquadrate petiole with a broadly rounded anterodorsal corner, rugose meso- and metapleural, head dorsum punctate, with relatively small eyes (EL 0.16–0.18). This species could be mistaken for *L.grohli* due to the ribbing across the notopropodeal sulcus, punctate head dorsum and small eyes, but *L.rufida* is smaller, with a more sculptured meso- and metapleural, and flagellomere segment I is as long as flagellomere segment II, shorter clypeal median lobe length (CML 0.19–0.21), and a petiole that is as high as long in lateral view (LPI 82.61–114.29), and as wide as long in dorsal view (DPI 76.76–90.83).

Across the Indochinese *Leptogenys* fauna, *L.rufida* is most similar to members of the *L.zhuangzii* species group (*L.mengzii* Xu, 2000, *L.laozii* Xu, 2000, and *L.zhuangzii* Xu, 2000), and can be differentiated by the following combined characters; petiole in lateral view as long as high, anterodorsal margin distinctly convex, sculptured meso- and metapleural, and red colour. *Leptogenysrufida* and *L.confucii* can be differentiated by the triangular shaped petiole of *L.confucii*, the smaller eyes in *L.rufida* and the densely sculptured meso- and metapleural of *L.confucii*.

##### Distribution.

*Leptogenysrufida* is known from China only, including the Chinese provinces of Guangxi, Yunnan, Zhejiang and now Hong Kong SAR (Zhou et al. 2012; Xu and He 2015). To our knowledge, this species is not reported from other southern Chinese provinces but considering the gap in records between Yunnan, Guangxi and Zhejiang, it seems likely to be found in Guangdong and Fujian.

##### Ecology.

Records for *L.rufida* in Hong Kong are sparse, but when collected it has occurred predominantly within pitfall traps, leaf litter samples and hand collection events (predominantly within leaf litter or soil) from secondary forest habitats. Two colony collections are known from Hong Kong. One colony was located within a half-soil filled metallic pipe buried within leaf litter, consisting of ~ 20 workers and one ergatoid queen, but was not collected. A second nest was located beneath a small rock, with the colony located ~ 4–5 cm below the upper soil layer (MTH403). The colony consisted of one male, one ergatoid, and nine workers. In addition, the latter colony was retained for dietary assessment with workers responding and feeding upon isopods and termites. Foraging workers in the latter colony’s collection locality were observed moving within leaf litter, with one worker returning with an isopod held ventrally between the legs.

##### Material examined.

Workers (*n* = 13): China • 1 worker; Hong Kong SAR, Pok Fu Lam; 26 Jun. 1993; J.R.Fellowes leg.; HKBM MBS015252. • 1 worker; Hong Kong SAR, Tai Mo Shan, Central NT; 20 Aug. 1993; J.R.Fellowes leg.; HKBM MBS015253. • 1 worker; Hong Kong SAR, Tai Po Kau; 20 May. 1993; J.R.Fellowes leg.; HKBM MBS015251. • 1 worker; Hong Kong SAR, The Peak; 24 Sep. 1993; J.R.Fellowes leg.; IBBL MBS006585. • 1 worker; Hong Kong SAR, Castle Peak; 22.38993, 113.95493; 426 m a.s.l.; 30 Jun. 2015; R.H.Lee leg.; Secondary forest, Pitfall trap; IBBL RHL01259. • 1 worker; Hong Kong SAR, Lin Fa Shan; 22.3956, 114.0885; 480 m a.s.l.; 15 Jul. 2016; R.H.Lee leg.; Plantation, Pitfall trap; IBBL RHL003291. • 1 worker; Hong Kong SAR, Guildford Road; 22.26715, 114.16248; 280 m a.s.l.; 18 Apr. 2018; C.Y.L.Tse leg.;, Hand collection. Leaf litter; IBBL ANTWEB1010123. • 4 workers; Hong Kong SAR, Tai Mo Shan; Kap Lung Forest Trail; 22.41088, 114.10451; 450 m a.s.l.; 3 Sep. 2023; M.T.Hamer leg.; Secondary forest, un. Rock; IBBL MTH403, ANTWEB1010205, ANTWEB1010232, ANTWEB1010233, ANTWEB1010234. • 1 worker; Hong Kong SAR, Tai Mo Shan; Kap Lung Forest Trail; 22.41088, 114.10451; 450 m a.s.l.; 17 Sep. 2023; M.T.Hamer leg.; Secondary forest, Gen. forager; IBBL ANTWEB1010202. • 1 worker; Hong Kong SAR, Tai Mo Shan; Kap Lung Forest Trail; 22.41088, 114.10451; 450 m a.s.l.; 17 Sep. 2023; M.T.Hamer leg.; Secondary forest, Winkler; IBBL ANTWEB1010179.

***Paratype*** ergatoid (*n* = 1): China • 1 ergatoid; Hong Kong SAR, Tai Mo Shan; Kap Lung Forest Trail; 22.41088, 114.10451; 450 m a.s.l.; 3 Sep. 2023; M.T.Hamer leg.; Secondary forest, un. Rock; IBBL ANTWEB1010234.

#### ﻿ Leptogenys strena

Taxon classificationAnimaliaHymenopteraFormicidae

Zhou, 2001

3D8481B9-F260-5B74-A024-A826CA63A7F3

**Figure 19.**
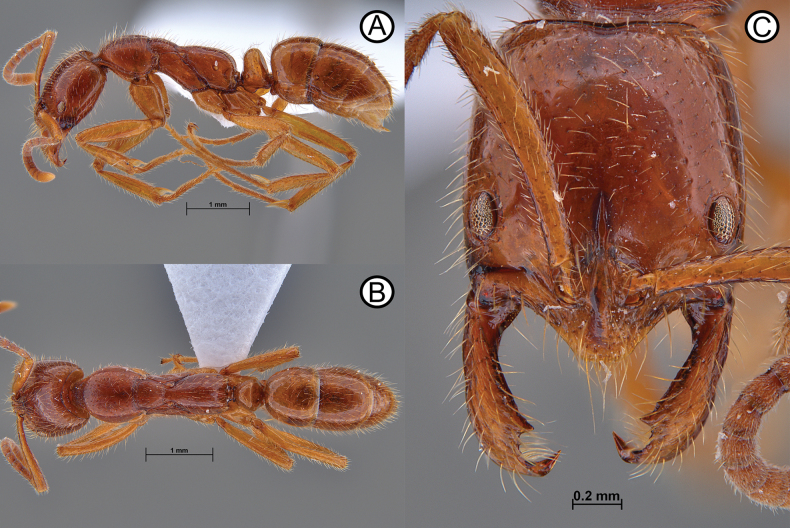
*Leptogenysstrena* (ANTWEB1010114) **A** lateral view **B** dorsal view **C** head in full face view.

Figs 4A , 19 , 21E new record

Leptogenys strena Zhou, 2001a: 40, 229, figs 39, 40 (w.) China (Guangxi).

##### Measurements.

Workers (*n* = 8): HL 1.39–1.47; HLL 1.16–1.23; HLA 0.23–0.31; HW 1.16–1.25; CML 0.21–0.26; SL 1.08–1.18; AII 0.2–0.25; AFI 0.18–0.22; AFII 0.15–0.19; EL 0.19–0.23; ML 0.72–0.8; PrL 0.85–0.95; PrH 0.59–0.64; PrW 0.82–0.99; WL 2.18–2.37; PeL 0.34–0.5; PeH 0.72–0.78; PeW 0.46–0.54; DPL 0.2–0.28; CI 82.86–87.03; CLI 15.38–17.74; SI 91.44–97.06; OI 16.6–18.83; LPI 144.33–216.28; DPI 166.19–249.29.

##### Morphological variation.

Upon examination of five specimens from the only collection locality in Hong Kong, it was noted that the number of teeth on the masticatory margin are not consistent but were always between three and five. Teeth were either conspicuous or barely discernible from the mandible margin, suggesting these teeth might be worn down and, or broken. A distinct diastema between the basal tooth and fourth tooth was, however, always present. The utility of teeth count as a differentiable and diagnosable character should likely be treated with caution unless combined with additional less variable characters.

**Figure 20.**
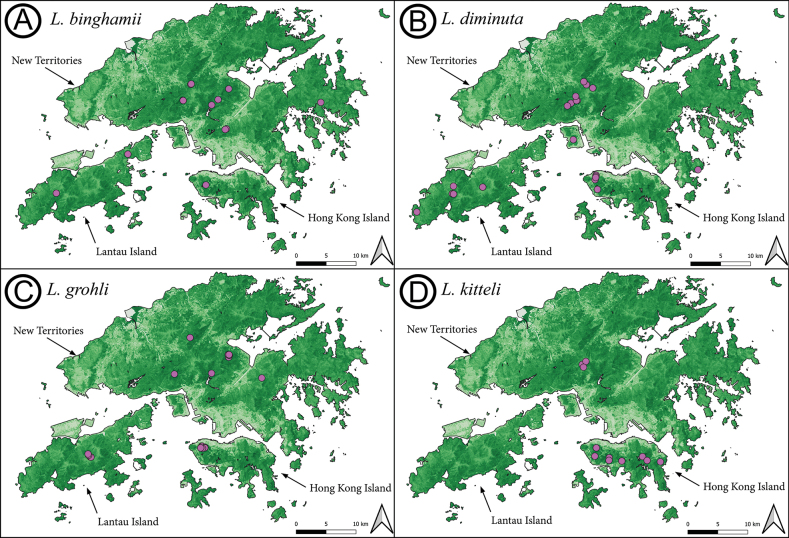
Distribution maps of *Leptogenys* species recorded from Hong Kong **A***L.binghamii***B***L.diminuta***C***L.grohli***D***L.kitteli*. The base map displayed shows tree canopy cover with the darker green areas indicating greater tree cover.

**Figure 21.**
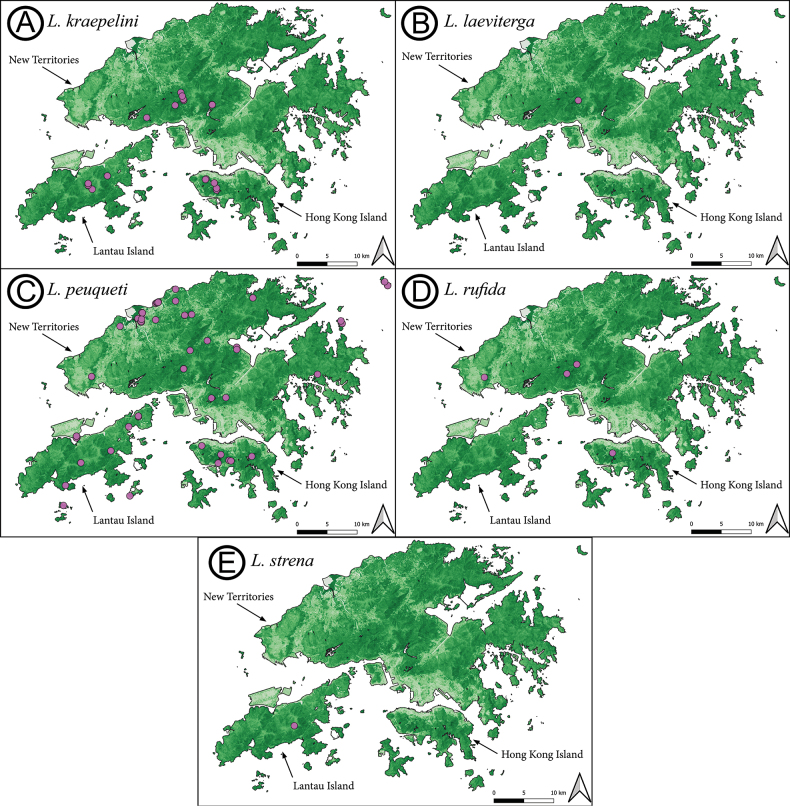
Further distribution maps of *Leptogenys* species recorded from Hong Kong **A***L.kraepelini***B***L.laeviterga***C***L.peuqueti***D***L.rufida***E***L.strena*. The base map displayed shows tree canopy cover with the darker green areas indicating greater tree cover.

##### Comparative notes.

*Leptogenysstrena* is immediately recognisable within the *Leptogenys* of Hong Kong by the combined characters of small anteriorly positioned eyes, a subquadrate head, triangular mandibles with 3–5 teeth on the masticatory margin, and a distinctly red body colouration. Within the wider *Leptogenys* fauna of China, *L.strena* is most similar to *L.lucidula* Emery, 1895 and can be differentiated by the rounded posteroventral corner of the subpetiolar process, fewer teeth on the masticatory margin and the species overall larger size.

##### Distribution.

This species is so far only known from China, including Guangxi (type locality), Hunan, and Guangdong, and Hong Kong SAR (Zhou et al. 2012; Xu and He 2015). Absence from other southern Chinese provinces is likely attributable to sampling effort.

##### Ecology.

Next to nothing is known about the ecology of *L.strena*. Specimens from Hong Kong are known only from Sunset Peak, Lantau Island. One collection was by J. R. Fellowes in 1992, and another in 2023 by A. Reshchikov using a ground SLAM trap, which was positioned in a tiny fragment of forest near the top of the mountain which yielded one further specimen. Further sampling efforts by members of Insect Biodiversity and Biogeography Laboratory have yet to collect additional specimens from this locality and from Hong Kong at large. The species’ small eyes might suggest leaf litter and/or nocturnal foraging.

##### Material examined.

Workers (*n* = 13): China • 8 workers; Hong Kong SAR, Lantau, Sunset Peak; 8 Oct. 1996; J.R.Fellowes leg.; HKBM MBS015294, MBS006591, MBS006592, ANTWEB1010116, ANTWEB1010115, ANTWEB1010114, ANTWEB1010167, ANTWEB1010166. • 4 workers; Guangdong Prov., Bai Yong; 3 May. 1998; J.R.Fellowes leg.; HKBM ANTWEB1010177, ANTWEB1010178, MBS006593. • 1 worker; Hong Kong SAR, Sunset Peak; 22.26104, 113.96683; 625 m a.s.l.; 06–25 May 2023; A. Reshchikov leg.; Secondary forest, SLAM ground; IBBL ANTWEB1010228.

## ﻿Discussion

Considering the relatively small geographical size (1100 km^2^), the genus *Leptogenys* is surprisingly rich in Hong Kong. Nine species are now recorded, including one new species, *L.grohli* sp. nov. Hong Kong is now the third most diverse region for this genus across Mainland China and Taiwan even with additional species provided for Guangdong, a considerably larger Mainland Chinese province (Table 1). Such low diversity for Guangdong, as well as Fujian and the more tropical Chinese Province of Hainan (Table 1), is undoubtedly attributable to limited sampling and taxonomic efforts, and is unlikely to reflect true biodiversity patterns. The lack of regional knowledge, especially for a large, mostly epigaeic, genus such as *Leptogenys*, indicates our lack of knowledge regarding the biodiversity of this region, a pattern also observed in other recently reviewed ant genera (e.g., *Polyrhachis*: Wong and Guénard 2021; *Ponera*: Leung et al. 2023; *Stigmatomma* and *Prionopelta*: Hamer et al. 2023a; *Strumigenys*: Tang and Guénard 2023).

**Table 1.** Distributional checklist of the *Leptogenys* species for the provinces of Mainland China and Taiwan. NR indicates new records presented in this study.

Species	Fujian	Guangdong	Guangxi	Guizhou	Hainan	Hong Kong	Hubei	Hunan	Jiangxi	Macao	Sichuan	Taiwan	Xizang	Yunnan	Zhejiang
*Leptogenysbinghamii* Forel, 1900			✓			NR								✓	
*Leptogenysbirmana* Forel, 1900					✓									✓	
*Leptogenyschinensis* (Mayr, 1870)	✓	✓	✓	✓				✓		✓	✓	✓		✓	
*Leptogenysconfucii* Forel, 1912												✓			
*Leptogenyscrassicornis* Emery, 1895														✓	
*Leptogenysdavydovi* Karavaiev, 1935														✓	
*Leptogenysdiminuta* (F. Smith, 1857)	✓	✓	✓		✓	✓		✓				✓		✓	
*Leptogenysgrohli* Hamer et al., sp. nov.		NR				✓									
*Leptogenyshainanensis* Chen et al., 2024					✓										
*Leptogenyshezhouensis* Zhou, 2001			✓												
*Leptogenyshuapingensis* Zhou, 2001			✓												
*Leptogenyskitteli* (Mayr, 1870)	✓	✓	✓	✓	✓	✓	✓	✓	✓		✓	✓		✓	✓
*Leptogenyskraepelini* Forel, 1905						✓								✓	
*Leptogenyslaeviterga* Zhou et al., 2012			✓			NR									
*Leptogenyslaozii* Xu, 2000														✓	✓
*Leptogenyslucidula* Emery, 1895														✓	
*Leptogenysmengzii* Xu, 2000													✓	✓	
*Leptogenyspangui* Xu, 2000														✓	
*Leptogenyspeuqueti* (André, 1887)	✓	✓	✓		✓	✓	✓	✓		✓				✓	✓
*Leptogenysprocessionalis* (Jerdon, 1851)														✓	
*Leptogenyspunctiventris* (Mayr, 1879)					✓										
*Leptogenysrufida* Zhou et al., 2012			✓			NR								✓	✓
*Leptogenysstrena* Zhou, 2001		NR	✓			NR		✓							
*Leptogenyssunzii* Xu & He, 2015														✓	
*Leptogenysyandii* Xu & He, 2015													✓		
*Leptogenyszhoui* Chen et al., 2024					✓										
*Leptogenyszhuangzii* Xu, 2000														✓	
**Total**	**4**	**6**	**10**	**2**	**7**	**9**	**2**	**5**	**1**	**2**	**2**	**4**	**2**	**17**	**4**

Species of *Leptogenys* are predominately collected via pitfall trapping and hand collection, with fewer known from Winkler samples, at least from Hong Kong. This is likely a result of their more epigaeic, nomadic, and nocturnal nature, at least for a few species. Some species seem to be locally rare, including *L.rufida*, *L.strena*, and *L.laeviterga*, which are known from just a few localities. *Leptogenysstrena* and *L.laeviterga* are known from a single locality from Sunset Peak, Lantau Island, and Tai Mo Shan, New Territories. Considering the sampling effort carried out across Hong Kong, comprising thousands of pitfall traps, Winkler samples and hand searching events, it is surprising that more material from more localities has not been collected, suggesting that both species are scarce.

Although Xu and He (2015) provided a comprehensive review of the genus in the Oriental region, much is yet to be understood about the taxonomy of the genus in Asia. For example, several species groups have been proposed, based upon limited morphological evidence (but see Arimoto 2017; Arimoto and Yamane 2018). Moreover, several species complexes (e.g., those of *L.diminuta*, *L.kitteli*, and *L.kraepelini*) exist within the region (Wilson 1958; Xu and He 2015). In addition, many regions lack sampling efforts including West and East Malaysia, Vietnam, Laos, and Cambodia. Thus, with known uncertain species groups, new species to science, unsorted species complexes, and lack of regional revisions, the *Leptogenys* is clearly in desperate need of greater taxonomic attention.

## Supplementary Material

XML Treatment for Leptogenys binghamii

XML Treatment for Leptogenys diminuta

XML Treatment for Leptogenys grohli

XML Treatment for Leptogenys kitteli

XML Treatment for Leptogenys kraepelini

XML Treatment for Leptogenys laeviterga

XML Treatment for Leptogenys peuqueti

XML Treatment for Leptogenys rufida

XML Treatment for Leptogenys strena

## ﻿Additional information

### Author ORCIDs

Matthew T. Hamer https://orcid.org/0000-0001-6728-9046

Jonathan Hon Chung Lee https://orcid.org/0009-0006-0204-8990

Cheung Yau Leo Tse https://orcid.org/0009-0005-7864-2198

Thiago S. R. Silva https://orcid.org/0000-0002-4239-1500

Benoit Guénard https://orcid.org/0000-0002-7144-1175

### Data availability

All of the data that support the findings of this study are available in the main text or Supplementary Information.

## Supplementary materials

Supplementary material 1 *Leptogenys* of Hong Kong measurements Data type xlsx Explanation note Measurements used to construct measurement ranges. https://binary.pensoft.net/file/1051516This dataset is made available under the Open Database License (http://opendatacommons.org/licenses/odbl/1.0/). The Open Database License (ODbL) is a license agreement intended to allow users to freely share, modify, and use this Dataset while maintaining this same freedom for others, provided that the original source and author(s) are credited.Matthew T. Hamer, Jonathan Hon Chung Lee, Cheung Yau Leo Tse, Thiago S. R. Silva, Benoit Guénard
